# Targeting the intestinal circadian clock by meal timing ameliorates gastrointestinal inflammation

**DOI:** 10.1038/s41423-024-01189-z

**Published:** 2024-06-25

**Authors:** Yunhui Niu, Marjolein Heddes, Baraa Altaha, Michael Birkner, Karin Kleigrewe, Chen Meng, Dirk Haller, Silke Kiessling

**Affiliations:** 1 ZIEL - Institute for Food & Health, Technical University of Munich, 85354 Freising, Germany; 2 Chair of Nutrition and Immunology, School of Life Sciences, Technical University of Munich, Gregor-Mendel-Str. 2, 85354 Freising, Germany; 3 Bavarian Center for Biomolecular Mass Spectrometry, Technical University of Munich, Gregor-Mendel-Str. 4, 85354 Freising, Germany; 4 Faculty of Health and Medical Sciences, University of Surrey, Stag Hill Campus, GU27XP Guildford, UK

**Keywords:** Intestinal circadian clock, Gastrointestinal inflammation, IBD, Restricted feeding, Microbiota, Mucosal immunology, Mechanisms of disease

## Abstract

The expression of clock genes has been observed to be impaired in biopsies from patients with inflammatory bowel disease (IBD). Disruption of circadian rhythms, which occurs in shift workers, has been linked to an increased risk of gastrointestinal diseases, including IBD. The peripheral circadian clock in intestinal epithelial cells (IECs) was previously shown to balance gastrointestinal homeostasis by regulating the microbiome. Here, we demonstrated that the intestinal clock is disrupted in an IBD-relevant mouse model (*IL-10*^−/−^). A lack of the intestinal clock gene (*Bmal1*) in intestinal epithelial cells (IECs) in a chemically and a novel genetically induced colitis model (DSS, *Bmal1*^IEC−/−^x*IL-10*^−/−^) promoted colitis and dramatically reduced survival rates. Germ-free *Bmal1*^IEC−/−^ mice colonized with disease-associated microbiota from *IL-10*^−/−^ mice exhibited increased inflammatory responses, highlighting the importance of the local intestinal clock for microbiota-induced IBD development. Targeting the intestinal clock directly by timed restricted feeding (RF) in *IL-10*^−/−^ mice restored intestinal clock functions, including immune cell recruitment and microbial rhythmicity; improved inflammatory responses; dramatically enhanced survival rates and rescued the histopathological phenotype. In contrast, RF failed to improve IBD symptoms in *Bmal1*^IEC−/−^x*IL-10*^−/−^ mice, demonstrating the significance of the intestinal clock in determining the beneficial effect of RF. Overall, we provide evidence that intestinal clock dysfunction triggers host immune imbalance and promotes the development and progression of IBD-like colitis. Enhancing intestinal clock function by RF modulates the pathogenesis of IBD and thus could become a novel strategy to ameliorate symptoms in IBD patients.

## Introduction

The circadian (lat. circa = approximately, dies = day) system consists of the central pacemaker in the suprachiasmatic nucleus (SCN), which drives behavioral rhythms and orchestrates peripheral clocks [1]. Disruption of the circadian system, for example, as experienced during jet lag [2], is related to immune deficits and pathologies, such as cancer and infections [3, 4], and has been associated with a wide range of diseases, including inflammatory bowel disease (IBD) [5].

As previously shown, tissue-specific functions are controlled by local peripheral clocks [2, 6]. Importantly, circadian rhythms, including those related to mucosal immunity and the microbiome, have been identified in the intestine [7–9]. Disturbance of the gastrointestinal (GI) physiology results in changes in the microbiota composition, which plays a role in the development of GI diseases, including IBD [10]. Our recent work highlights the importance of functional intestinal clocks for host–microbe crosstalk to balance GI homeostasis [11]. Hence, a local intestinal oscillator may impact the development of GI disorders. Indeed, epidemiological studies have reported suppressed clock gene expression in biopsies from IBD patients [12], indicating a link between the intestinal clock and disease occurrence. However, the role of the intestinal clock in GI diseases remains to be addressed. Here, we hypothesized that the intestinal clock may constitute a mechanism by which mucosal immune functions and the microbiome promotes GI inflammation.

Recently, time-restricted eating/feeding (RF) has become increasingly popular due to its positive effects on metabolic health and inflammation and its impact on the gut microbiome [13]. Preclinical evidence showed that RF improves systemic inflammation [14]. RF is also a powerful tool for regulating peripheral clocks [15], including in the intestinal tissues [9], and microbial rhythmicity [8]. Consequently, we hypothesize that RF may influence the development and progression of IBD by targeting intestinal clock functions.

In this study, we demonstrated the causal relationship between intestinal clock dysfunction and the severity of GI inflammation in chemically and genetically induced colitis models (*IL-10*^−/−^, *Bmal1*^IEC−/−^x*IL-10*^−/−^, dextran sulfate sodium (DSS)) and microbiota transfer experiments. Potential mechanisms include regulation of key genes relevant for inflammatory and bacterial responses by the intestinal clock, which were identified by RNA-sequencing analysis. Importantly, for the first time, we demonstrated the beneficial effects of nighttime-restricted feeding (RF) on circadian colon functions, the microbiome, IBD progression and survival of *IL-10*^−/−^ mice, a well-described mouse model of colitis [16]. Remarkably, RF failed to ameliorate colitis symptoms and improve survival in intestinal clock-deficient *Bmal1*^IEC−/−^x*IL-10*^−/−^ mice, demonstrating the relevance of a functional intestinal clock in mediating the positive effect of RF; thus, RF might be a novel target for future IBD therapies.

## Results

### Intestinal circadian disruption in an IBD-relevant mouse model

**Fig. 1:**
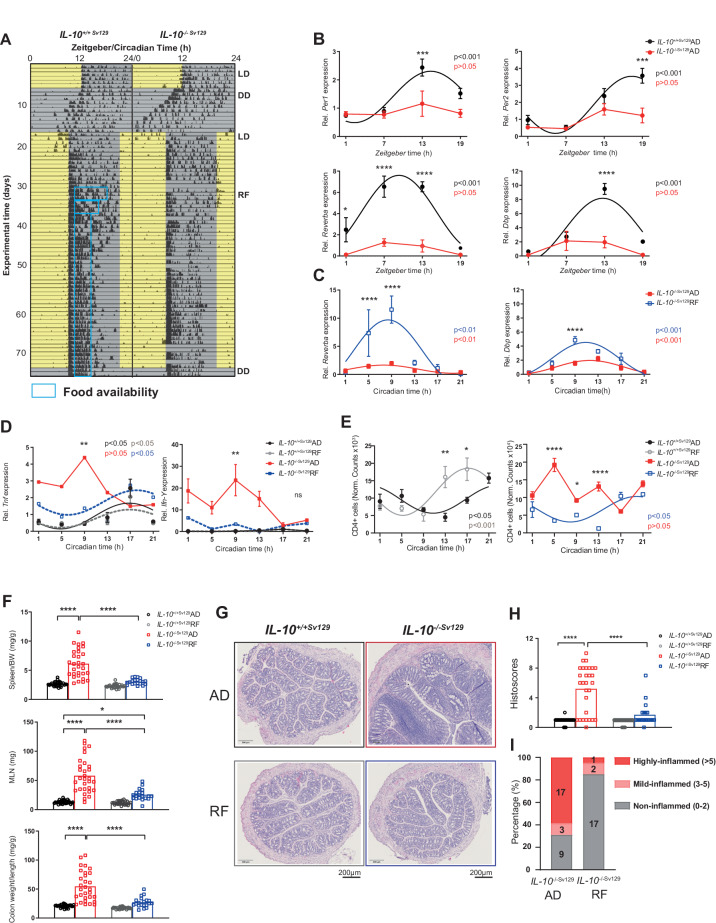
Restricted feeding restores the disrupted colonic clock in *IL-10*^−/−Sv129^ mice and ameliorates colitis. **A** Representative actogram of wild type (*IL-10*^+/+Sv129^) and *IL-10*^−/−Sv129^ mice exposed to a light (yellow)-dark (gray) cycle (LD), constant darkness (DD), and nighttime-restricted feeding (RF, blue boxes). Tick marks represent running wheel activity. **B** Clock gene expression in colonic tissues from *IL-10*^−/−Sv129^ mice (Control AD and RF: *n* = 24; *IL-10*^−/−Sv129^AD: *n* = 24; *IL-10*^−/−Sv129^RF: *n* = 19; *n* = 3-4 mice/time points for each group) **C** Clock gene and **D** inflammatory genes expression in colonic tissues. **E** Quantification of the amount of CD4 + T cells in the colon LP from wild type and *IL-10*^−/−Sv129^ mice under AD and RF conditions. **F** Organ weights, **G** representative colonic cross section stained with H&E and **H** histopathological scores of colonic tissues from wild-type and *IL-10*^−/−Sv129^ mice under AD and RF conditions (top) and **I** quantification of individual mice with different degrees of inflammation (bottom). Tissues with scores between 0 and 2 were classified as noninflamed, those with scores between 3 and 5 were classified as mildly inflamed, and those with scores >5 were classified as highly inflamed. The data points represent individual mice (*n* = 3–4/time point for each group). Significant rhythms are illustrated with fitted cosine-wave regression using a line (significance: cos-fit *p* value ≤ 0.05). Two-way ANOVA with Tukey’s correction was also used. The data are presented as the means ± SEMs. Asterisks indicate significant differences; *p < 0.05, ***p < 0.001, ****p < 0.0001. Details on the number of mice per time point are summarized in the Supplementary Table 6

The development of various GI diseases, including IBD [5], has been linked to circadian disruption, such as that occurring during shift work and frequent travel across time zones [2]. *IL-10-*deficient mice on a Sv129 background (*IL-10*^−/− Sv129^) exhibit a normal circadian behavioral phenotype (Fig. 1A, Supplementary Fig. 1A). Central circadian clock functions, such as rhythmic wheel-running activity and food intake during a light–dark (LD) cycle or constant darkness (DD), were indistinguishable from those of the controls, although total activity was slightly reduced in the *IL-10*^−/− *Sv129*^ mice (Fig. 1A, Supplementary Fig. 1B–D).

Recently, we demonstrated an imbalance in GI immune homeostasis in mice lacking a functional intestinal clock [11]. This prompted us to characterize the molecular intestinal clock in inflamed *IL-10*^−/− Sv129^ mice. Robust circadian rhythms were found in the expression of the clock genes *Per1/2* (*Period 1/2*), *Rev-erbα* (*NR1D1*) and *Dbp* in the colonic epithelial cells of wild-type mice. This rhythmicity was lost, and gene expressions were strongly suppressed in *IL-10*^−/− *Sv129*^ mice (Fig. 1B), indicating a dysfunctional colonic clock. The circadian origin of these results was validated in colonic tissues collected at 6 different circadian times from *IL-10*^−/− *Sv129*^ mice kept in constant darkness and in cultured colonic organoids (Supplementary Fig. 1E, F). This effect was restricted to the colon since the major clock genes remained rhythmic in the liver, cecum and cultured jejunal organoids (Supplementary Fig. 2). Loss of rhythmicity in *IL-10*^−/− *Sv129*^ mice was also observed for inflammatory genes, e.g., *Tnf, Ifnγ, Cxcr4*, and *Vcam-1*, and T-cell recruitment to the lamina propria (LP) (Fig. 1D, E, Supplementary Fig. 1G, H). Furthermore, the peak expression of most clock genes was strongly correlated with the colonic histological score; the frequency of immune cells in the lamina propria, including macrophages, neutrophils, CD4^+^ cells and dendritic cells; and the expression of genes involved in barrier function (Supplementary Fig. 1I, J). These results indicate a causal relationship between colon clock dysfunction in *IL-10*^−/− *Sv129*^ mice and their inflammatory phenotype.

### Nighttime-restricted feeding restored the colonic clock and ameliorated pathological changes in *IL-10*^−/−^ mice

Nighttime-restricted feeding (RF) is a widely used approach in mice to influence the rhythmicity of peripheral clocks [15]. We tested whether RF in *IL-10*^−/− Sv129^ mice restores the colonic clock and impacts their Crohn’s disease-like phenotype. Therefore, RF was gradually introduced to *IL-10*^−/− Sv129^ mice and controls, which were euthanized after 4 weeks of RF on 6 different circadian times (Fig. 1A, Supplementary Fig. 1A). Food intake and nighttime activity levels were comparable between genotypes and feeding conditions (Supplementary Fig. 1B–D). Importantly, RF substantially improved the circadian rhythmicity of clock gene expression in *IL-10*^−/− Sv129^ colonic tissue to similar levels as those observed in control mice under *ad libitum* (AD) conditions (Fig. 1C, Supplementary Table 1). Moreover, a restored rhythmicity of *Tnf* and reduced levels of *Tnf* and *Ifn-y* as well as a restored rhythmicity in the number of CD3^+^CD4^+^ cells isolated from the colonic LP and an overall reduced number of CD4 + T lymphocytes were observed in *IL-10*^−/− *Sv129*^ mice during RF in comparison to those in AD-fed *IL-10*^−/− *Sv129*^ mice (Fig. 1D, E, **right**). In contrast, CD3^+^CD8^+^ cell recruitment remained arrhythmic (Supplementary Fig. 1H). Consistently, the weights of the spleen and mesenteric lymph nodes (MLNs) and the colon density, which were much heavier in the AD-fed *IL-10*^−/− *Sv129*^ mice than in the control mice, were significantly lower during RF and were undistinguishable from those of the controls (Fig. 1F). Histological analysis of colon cross-sections further revealed that more than 58% of AD-fed *IL-10*^−/−*Sv129*^ mice were highly inflamed (Histoscore>5), which was dramatically reduced to 5% by RF (Fig. 1G–I). These data demonstrate that RF restores the disruption of colon clock function and completely ameliorates the immune phenotype in *IL-10*^−/−*Sv129*^ mice.

### Loss of microbial rhythmicity during colonic inflammation *in IL-10*^−/−^ mice is restored by RF

**Fig. 2:**
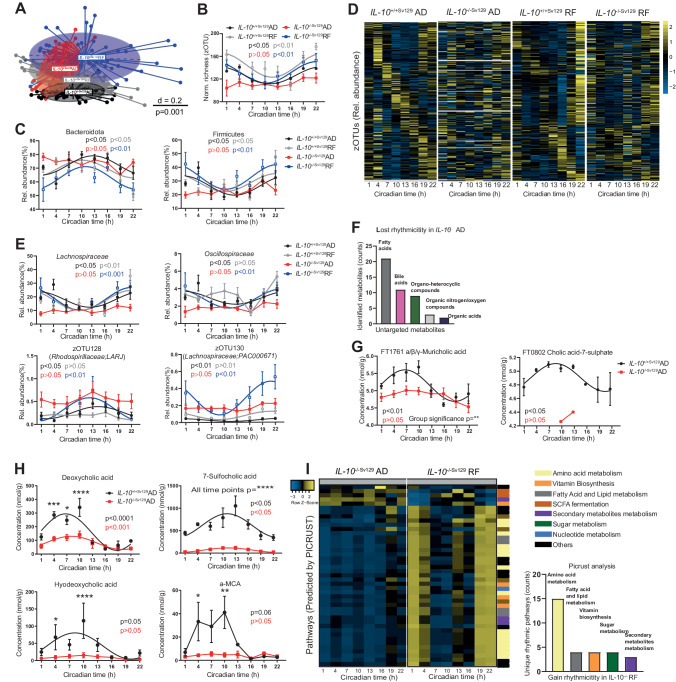
Impaired microbiome rhythms in *IL-10*^−/−SV129^ mice are restored after RF. **A** Beta-diversity illustrated by MDS plots of fecal microbiota based on generalized UniFrac distances (GUniFrac) in wild type and *IL-10*^−/−Sv129^ mice under AD and RF conditions. Comparison of genotype and feeding effects was performed by PERMANOVA (adj.*p* = 0.001). Data points represent individual sample collected from different mice at different time points (*n* > 5 mice/time points for each group). **B** Circadian profile of normalized richness and **C** relative abundance of phyla in wild type and *IL-10*^−/−Sv129^ mice under AD and RF conditions. **D** Heatmap depicting the relative abundance of zOTUs (mean relative abundance>0.1%; prevalence > 10%). Data from *IL-10*^−/−Sv129^ mice under AD or RF conditions are normalized to the peak of each zOTU and ordered by the peak phase in wild type mice under AD or RF conditions, respectively. Yellow and blue indicate high and low abundance, respectively. Each column represents the group average for each time point. **E** Relative abundance of highly abundant families and representative zOTUs from wild type and *IL-10*^−/−Sv129^ mice under AD and RF conditions. **F** Classification of the top 5 fully annotated super classes of metabolites that lost rhythmicity in *IL-10*^−/−Sv129^ mice under AD. Circadian profiles of bile acids (BAs) **G** measured by untargeted or **H** targeted metabolomics of wild type and *IL-10*^−/−Sv129^ mice under AD conditions. At some time points (CT 1, 4, 7, 16, 19, and 22) the levels for the IL-10−/− group were under the detection limit and thus not illustrated in the graph. **I** Heatmap of pathways (calculated by PICRUST 2.0) with restored rhythms in *IL-10*^−/−Sv129^ mice under RF (left) and the quantification of the superclass (right). Rhythmicity identified by JTK_Cycle (Bonferroni adj. *p* value ≤ 0.05). Significant rhythms are illustrated with fitted cosine-regression; data points connected by straight lines indicate no significant cosine fit curves (*p* > 0.05) and thus no rhythmicity. Comparisons of groups were performed by two-way ANOVA followed by Sidak correction. The results are summarized in Supplementary Table 3. Data are represented as the mean ± SEM. Details on mouse numbers per time point are summarized in Supplementary Table 6

Gut microbiota dysbiosis has been associated with the development of IBD in humans and mouse models [10]. Previously, we identified a novel link between the intestinal clock, microbiome rhythms and GI homeostasis [11]. Consistently, the microbiota composition differed significantly between genotypes despite the feeding conditions (Fig. 2A). Circadian rhythmicity in community diversity (normalized species richness), the abundance of both major phyla, Bacteroidota and Firmicutes, and highly abundant families, including *Lachnospiraceae* and *Oscillospiraceae*, as well as zOTUs, followed circadian oscillation in the wild type, whereas these rhythms were abolished in AD-fed *IL-10*^−/−Sv129^ mice (Fig. 2B–E). In accordance with the results obtained from IBD patients [17], alterations in the abundance of *Lachnospiraceae*, *Oscillospiraceae* and *Erysipelotrichaceae* were observed in *IL-10*^−/−^ mice (Supplementary Fig. 3A). The abundances of the genera *Oscillibacter, Eubacterium, Clostridium* and *Pseudoflavonifractor* significantly differed among the genotypes (Supplementary Fig. 3B**left**, Supplementary Table 2). Moreover, we detected significant correlations between disease markers, such as histological score, inflammatory marker gene expression and zOTUs belonging to *Lachnospiraceae* and *Oscillospiraceae* (Supplementary Fig. 3B**right**), which is consistent with our recent findings showing a correlation between *Lachnospiraceae* and active disease in IBD patients [18]. Importantly, RF not only restored the differences in abundance between genotypes (Supplementary Fig. 3B, Supplementary Table 3) but also restored microbial rhythmicity in *IL-10*^−/−Sv129^, as illustrated by the community diversity and at the levels of the major phyla and families (Fig. 2B–E). Enhanced microbial rhythmicity was also found among the 237 identified zOTUs visualized by heatmaps (Fig. 2D, Supplementary Fig. 3C). More than 50% of the zOTUs that gained rhythmicity during RF in *IL-10*^−/−^ mice belonged to the *Lachnospiraceae family* (Supplementary Table 2), which includes bacterial taxa important for SCFA production and secondary bile acid (BA) conversion and thus plays crucial roles in IBD progression [19, 20].

To address the physiological relevance of disturbed microbial oscillations in *IL-10*^−/−Sv129^ mice, (un)targeted metabolite analysis was performed on feces. Indeed, 55 fully annotated metabolites were clustered according to genotype (Supplementary Fig. 3D) in *IL-10*^−/−Sv129^ mice, most of which were lipids and lipid-like metabolites, including fatty acids and BAs (Fig. 2F). For example, the rhythmicity of bile acids, including muricholic acid and 7-sulfocholic acid, as well as the long-chain fatty acid oleic acid and linoleic acid, was lost and suppressed (Fig. 2G, Supplementary Fig. 3E). Targeted metabolomics further validated these genotype differences (Fig. 2H, Supplementary Fig. 3F). For example, in *IL-10*^−/−sv129^ mice, a highly suppressed amplitude or lost time difference was observed for primary and secondary bile acids (BAs), such as deoxycholic acid (DCA), hyodeoxycholic acid, 7-sulfocholic acid (7-sulfo-CA) and α-muricholic acid (Fig. 2H). Most of these secondary BAs are key mediators involved in gut dysbiosis, especially in IBD [21]. Additionally, reduced SCFA levels, such as those of acetate, butyrate and propionate, were found in *IL-10*^−/−sv129^ mice (Supplementary Fig. 3F), similar to observations in IBD patients and related mouse models [19]. The concentrations of valeric acid and desaminotyrosin were also suppressed in *IL-10*^−/−Sv129^ mice (Supplementary Fig. 3F). Importantly, PICRUSt2 analysis of samples obtained from *IL-10*^−/−Sv129^ mice under RF conditions revealed restored rhythmicity of the assigned pathways involved in fatty acid synthesis and SCFA fermentation (Fig. 2I). Taken together, these results indicate that the changes in microbial rhythmicity and composition observed in *IL-10*^−/−Sv129^ mice can be reversed by RF; thus, microbial rhythmicity might be involved in the beneficial effect of RF on colonic inflammation.

### Germ-free intestinal clock-deficient mice develop an increased inflammatory response following the transfer of *IL-10*^−/−^-associated microbiota

**Fig. 3:**
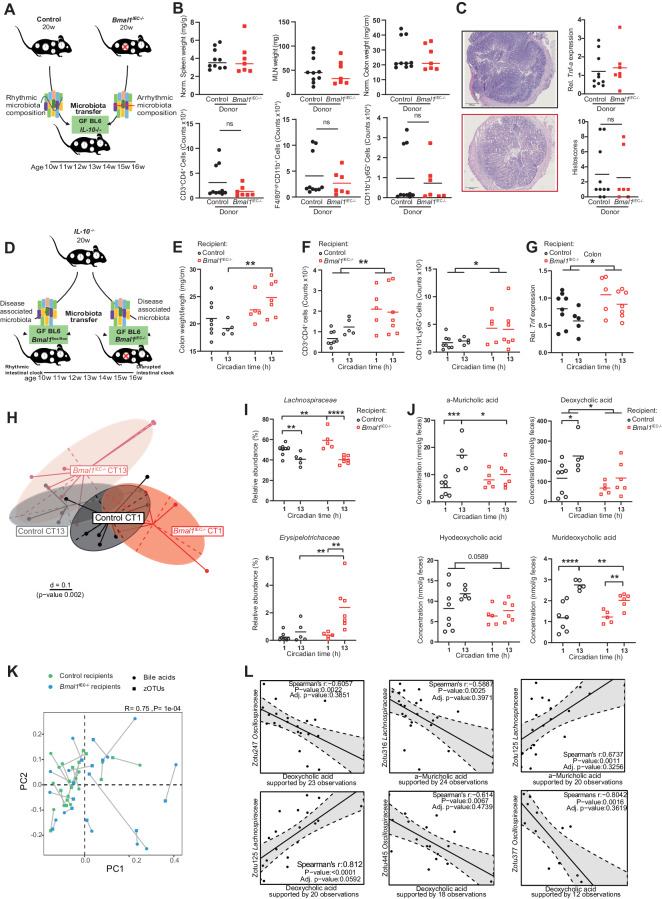
Dysfunction of the host intestinal clock promotes microbiota-induced colonic inflammation. **A** Schematic illustration of the experimental design. Cecal contents from control (rhythmic) and *Bmal1*^IEC−/−^ (arrhythmic) donors were transferred to germ-free *IL-10*^−/−BL6^ mice. **B** Organ weights (top) and number of immune cells recruited into the colonic LP (bottom) of the recipients. **C** Representative H&E staining of colonic cross sections from the recipients (left) and *Tnf* gene expression, as well as histopathological scores (right). **D** Schematic illustration of the experimental design. Disease-associated microbiota from *IL-10*^−/−^ donors was transferred to germ-free control (*Bmal1*^flox/flox^) and *Bmal1*^IEC−/−^ recipients. **E** Colon weight, **F** number of immune cells recruited into the colonic lamina propria and **G** *Tnf* expression in colon tissues from recipients. **H** Beta diversity illustrated by MDS plots of cecal microbiota based on generalized UniFrac distances (GUniFrac) in recipients at circadian times (CTs) 1 and 13. **I** Relative abundance of families and **J** amounts of α-muricholic acid (α-MCA), deoxycholic acid (DCA), hyodeoxycholic acid (HDCA) and murideoxycholic acid (MDCA) in the cecal contents of control and *Bmal1*^IEC−/−^ recipients. **K** Procrustes analysis (PA) of the cecal microbiota and bile acid (BA) levels. The length of the line is proportional to the divergence between the data from the same mouse. **L** Representative correlation plot between zOTUs and BAs. The data points represent individual mice (*n* > 5/time points for each genotype). Mann–Whitney U tests and two-way ANOVA followed by Benjamini–Hochberg correction were used. Asterisks indicate significant differences; *p < 0.05, **p < 0.01, ****p < 0.0001. The data are presented as the mean ± SEMs. Details on the number of mice per time point are summarized in Supplementary Table 6

Recently, we demonstrated that a functional intestinal clock is required to maintain GI homeostasis by driving the microbiome, including *Lachnospiraceae* and microbiota-derived DCA and 7-sulfo-CA [11]. To determine whether a dysfunctional intestinal clock in *IL-10*^−/−Sv129^ mice or the arrhythmicity of the microbiome directly affects the severity of inflammation, we performed two distinct cecal microbiota transfer experiments. First, colonialization of cecal content from intestine-specific clock-deficient (*Bmal1*^IEC−/−^) and littermate (*Bmal1*^flox/flox^) donors was performed to transfer rhythmic and arrhythmic microbiota into germ-free (GF) *IL-10*^−/−BL6^ recipients (Fig. 3A). Surprisingly, the weight of the immune organs and the amount of immune cell infiltration to the LP did not differ among the recipients (Fig. 3B). Consistently, histological staining, scoring, and inflammatory marker gene expression revealed no pathological differences between recipients (Fig. 3C), indicating that microbial rhythmicity might not directly induce intestinal inflammation. However, when GF *Bmal1*^IEC−/−^ mice received disease-associated microbiota from inflamed *IL-10*^−/−BL6^ mice, colon weight, immune cell recruitment to the colonic LP and *Tnf* gene expression were severely elevated in *Bmal1*^IEC−/−^ recipients compared to controls, although the histological score obtained from a single colon cross-section showed no clear difference between the recipients (Fig. 3D–G, Supplementary Fig, 4A–C). Interestingly, clustering between circadian time points and recipients was found for beta diversity in cecal samples (Fig. 3H). Alterations in the abundance of *Lachnospiraceae* and *Erysipelotrichaceae* in *Bmal1*^IEC−/−^ recipients were similar to those in *IL-10*^−/−Sv129^ mice (Fig. 3I, Supplementary Fig. 3A). Accordingly, the cecal concentrations of α-MCA, DCA, HDCA and MDCA were lower than those in control recipients (Fig. 3J, Fig. 2H). Procrustes analysis (PA) revealed an association between zOTUs and BA production; in particular, *Lachnospiraceae* and *Oscillospiraceae* were correlated with DCA and aMCA concentrations (Fig. 3K, L). Taken together, these results reflect an early stage of inflammation following colonization with disease-associated microbiota when recipients lack a functional intestinal clock. Consequently, the intestinal clock represents a functional link between the microbiome and GI inflammation.

### Genetic intestinal clock dysfunction alters colonic immune functions

**Fig. 4:**
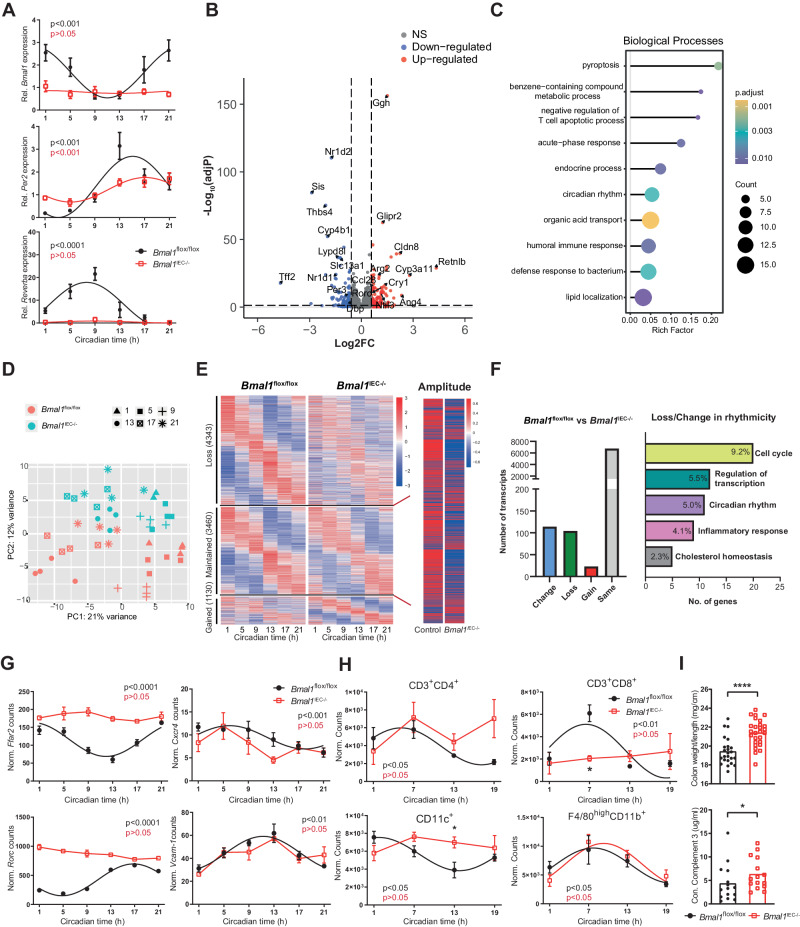
The intestinal clock regulates transcripts involved in intestinal immune responses. **A** Clock gene expression and **B** volcano plot of transcriptional changes (upregulated: adjusted *p* < 0.05, FC > 1.5, red; downregulated: adjusted *p* < 0.05, FC > 1.5, blue) in colon tissues from control and *Bmal1*^IEC−/−^ mice. **C** Gene Ontology enrichment analysis of differentially expressed genes. **D** Principal component analysis of 46 colon samples obtained from control (red) and *Bmal1*^IEC−/−^ (blue) mice. Different shapes indicate different circadian time (CT) points. **E** Heatmap of transcripts that lost, maintained or gained rhythmicity in colon tissues from *Bmal1*^IEC−/−^ mice (left) and amplitude comparison of the transcripts that maintained rhythmicity (right) (JTK_cycle adj.*p* < 0.05 as rhythmic). Transcripts are ordered according to the peak phase of the control groups. Each column represents the group average for each time point. **F** Number of transcripts whose rhythmic changes were lost/changed according to comparison (left) and GO analysis (top 5, right). Circadian profiles of **G** gene expression and immune cell frequency **H** in the colonic LP of control and *Bmal1*^IEC−/−^ mice. **I** Colon weight (top) and complement 3 levels in the feces of control and *Bmal1*^IEC−/−^ mice. The data points represent individual mice (*n* = 4/time points for each genotype). Mann–Whitney *U* test. Asterisks indicate significant differences; *p < 0.05, ****p < 0.0001. Significant rhythms are illustrated with fitted cosine-wave regression using a line (significance: cos-fit *p* value ≤ 0.05). Details on the number of mice per time point are summarized in Supplementary Table 6

To determine whether intestinal clock dysfunction influences the colonic immune response, we used *Bmal1*^IEC−/−^ mice. Colonic IEC clock dysfunction was validated by arrhythmic and suppressed clock gene expression (Fig. 4A). Furthermore, bulk RNA-sequencing analysis of colonic tissue samples obtained during the circadian day revealed differential expression between genotypes (fold change>1.5, adj. *p* < 0.05), such as *Retnlb* (Resistin-like beta) and *Tff*2 (Trefoil factor 2) (Fig. 4B), which are involved in colitis development [22, 23]. Consistently, gene ontology enrichment analysis highlighted pathways relevant for ‘humoral immune response,’ ‘defense response to bacterium’ and ‘T-cell apoptotic process’ (Fig. 4C). Clustering according to the genotype was observed by principal component analysis (PCA), but clustering according to the collection time point was less pronounced in *Bmal1*^IEC−/−^ (Fig. 4D). Consistently, 55% of rhythmic transcripts in the controls were arrhythmic in *Bmal1*^IEC−/−^ mice (Fig. 4E**left**, Supplementary Table 4). The majority of the resilient rhythmic transcripts showed a highly suppressed amplitude in *Bmal1*^IEC−/−^ mice (Fig. 4E, **right**). DODR analysis further confirmed that >200 genes either lost or changed rhythmicity (114/104, respectively) in *Bmal1*^IEC−/−^ mice and were enriched in pathways related to “circadian rhythm” and “inflammatory response” (Fig. 4F). In particular, genes involved in the inflammatory and immune responses [24–26], including *Ffar2*, *Hc, Abcc2* and *Rorc*, goblet cell function [27] and immune cell migration [28, 29], such as *Cxcr4* and *Vcam-1*, lost rhythmicity in *Bmal1*^IEC−/−^ mice (Fig. 4G, Supplementary Fig. 5A, B, Supplementary Table 4). Accordingly, immune cell frequency (CD4^+^ and CD8^+^ T cells and dendritic cells) lost rhythmicity in the colonic LP of *Bmal1*^IEC−/−^ mice (Fig. 4H), similar to the results obtained for *IL-10*^−/−Sv129^ mice (Fig. 1E). Although a lack of the intestinal clock did not result in a pathophysiological phenotype (Supplementary Fig. 5C–E), neither the colon weight nor the concentration of complement 3 in the stools of *Bmal1*^IEC−/−^ mice was significantly increased (Fig. 4I). These findings are consistent with the results obtained from *IL-10*^−/−^ mice (Fig. 1F, Supplementary Fig. 5F), suggesting that intestinal clock dysfunction might indeed magnify inflammatory processes in *IL-10*^−/−^ mice.

### *Bmal1*^IEC−/−^ mice are susceptible to acute and chronic colon inflammation

**Fig. 5:**
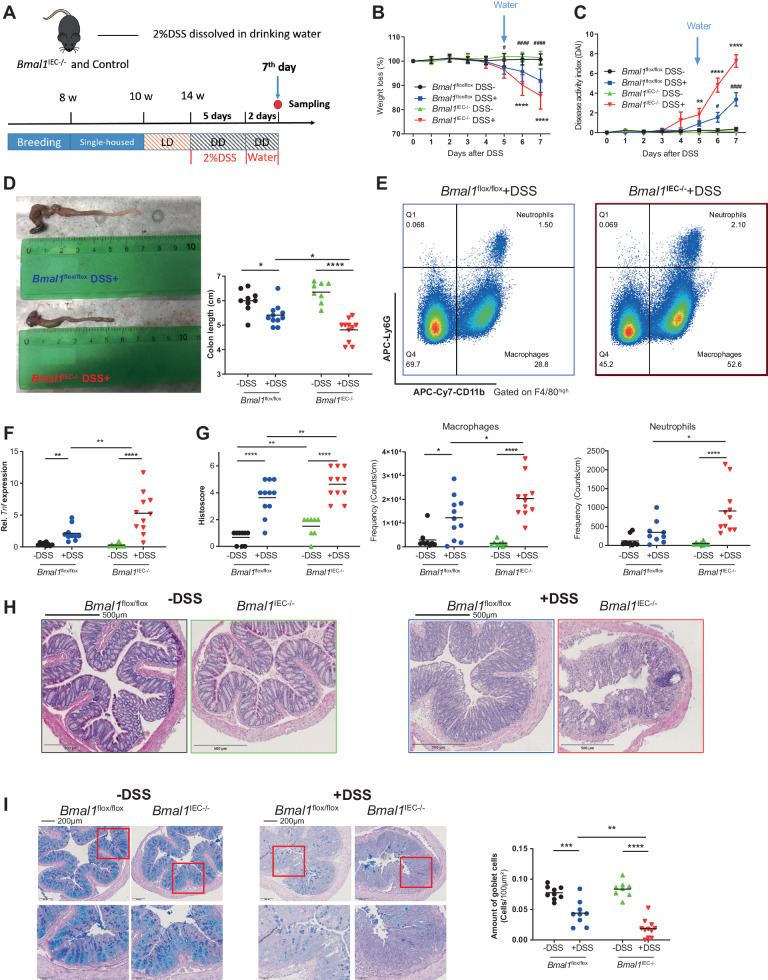
*Bmal1*^IEC−/−^ mice are more susceptible to DSS-induced colitis. **A** Schematic illustration of the DSS treatment procedure. Body weight changes **B** and disease activity indices **C** of control and *Bmal1*^IEC−/−^ mice during DSS/water administration. **D** Representative images of the colon (left) and colon length measurements (right). **E** Gating strategy for macrophages and neutrophils in the colon LP and quantification. *Tnf* expression in colon tissues **F**, histopathological scores **G**, representative H&E staining **H** and PAS-AB staining quantification of the number of goblet cells based on a minimum of 20 crypts for each section **I** of colon sections from control and *Bmal1*^IEC−/−^ mice with/without DSS treatment. The data points represent individual mice (*n* > 8 for each group). Two-way ANOVA and Benjamini‒Hochberg correction were used. Asterisks indicate significant differences between genotypes after DSS treatment; *p < 0.05, **p < 0.01, ***p < 0.001, ****p < 0.0001. Octothorpes indicate significant differences between *Bmal1*^flox/flox^ mice after treatment (#*p* < 0.05, ##*p* < 0.01, ###*p* < 0.001). Details on the number of mice per time point are summarized in Supplementary Table 6

To directly determine the cause‒effect relationship between intestinal clock dysfunction and GI inflammation, *Bmal1*^IEC−/−^mice and controls were released into DD condition and received drinking water supplemented with 2% DSS (Fig. 5A) to induce tissue damage and create a model of acute colitis [30]. DSS treatment of *Bmal1*^IEC−/−^ mice caused a significant reduction in body weight, an increase in the disease activity index (DAI) [31], and a decrease in colon length compared to DSS treated controls (Fig. 5B–D). Accordingly, the expression of inflammatory markers, such as *Tnf*, and neutrophil and macrophage recruitment to the colonic LP, were strongly enhanced in *Bmal1*^IEC−/−^ mice (Fig. 5E, F). Consistently, in the same mice, histopathological evaluation of colon cross-sections revealed significant differences between genotypes after DSS treatment, which was reflected by increased histological scores in *Bmal1*^IEC−/−^mice **(**Fig. 5G, H). Additionally, intestinal clock dysfunction caused the loss of goblet cells following DSS treatment (Fig. 5I), which represents a critical factor during DSS-induced colitis [31]. These results demonstrated increased sensitivity to DSS-induced colitis in *Bmal1*^IEC−/−^ mice.

**Fig. 6:**
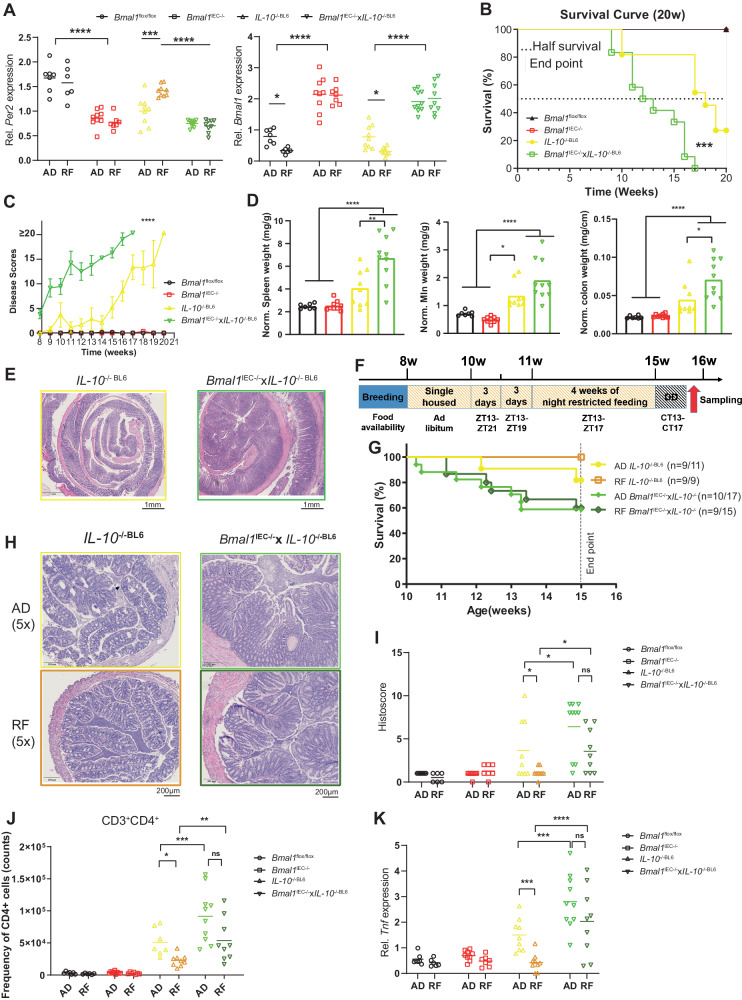
Restricted feeding ameliorates the IBD-like symptoms in mice by targeting the intestinal clock. Colon clock genes expression **A** and survival **B** in control, *Bmal1*^IEC−/−^, *IL-10*^−/−BL6^ and *Bmal1*^IEC−/−^x*IL-10*^−/−BL6^ mice under AD and RF conditions. Disease scores **C**, organ weights **D**, and representative H&E staining images of colon sections **E** from *IL-10*^−/−BL6^ and *Bmal1*^IEC−/−^x*IL-10*^−/−BL6^ mice under AD conditions. **F** Schematic illustration of the experimental RF design. Survival analysis **G**, representative H&E staining of colon sections **H**, histopathological scores **I**, immune cell frequency in the colonic LP **J** and *Tnf* expression **K** in *IL-10*^−/−BL6^ and *Bmal1*^IEC−/−^x*IL-10*^−/−BL6^ mice under AD and RF conditions. The data points represent individual mice (*n* > 6/time points for each group). One-way ANOVA, two-way ANOVA following Benjamini‒Hochberg correction, and three-way ANOVA followed by Benjamini–Krieger-–Yekutieli correction were used. *p < 0.05, **p < 0.01, ***p < 0.001, ****p < 0.0001. The data are presented as the mean ± SEM. Details on the number of mice per time point are summarized in Supplementary Table 6

To further assess the impact of the intestinal clock on IBD development, we evaluated IBD pathology in a newly generated genetic mouse model prone to developing chronic colitis combined with a dysfunctional intestinal clock (*Bmal1*^IEC−/−^x*IL-10*^−/−BL6^). IL-10 deficiency on the BL6 background causes less severe colitis symptoms than on the SV129 background [16], which is likely a result of less severe alterations in clock gene expression (Fig. 6A, Fig. 1B, Supplementary Fig. 6F). The additional loss of the intestinal clock dramatically reduced the survival rates of *Bmal1*^IEC−/−^x*IL-10*^−/−BL6^ mice (Fig. 6B). In particular, all *Bmal1*^IEC−/−^x *IL-10*^−/−BL6^ mice were euthanized before the end of the experiment due to their severe disease burden, while approximately 30% of *IL-10*^−/−BL6^ mice survived. More severe tissue inflammation was observed in *Bmal1*^IEC−/−^x*IL-10*^−/−BL6^ mice, which was reflected by increased spleen, colon and MLN weights and increased disease scores (Fig. 6C–E). Similar to our previous results obtained from *Bmal1*^IEC−/−^ mice [11], the microbiota diversity differed between genotypes, and circadian rhythmicity in terms of species richness and the abundance of the major phyla was dramatically disrupted in *Bmal1*^IEC−/−^x*IL-10*^−/−BL6^ mice, whereas only mild circadian changes were observed in *IL-10*^−/−BL6^ mice (Supplementary Fig. 6A–C). Moreover, a heatmap showing the peak relative abundances of zOTUs and their quantification confirmed the disruption of microbial rhythmicity in *Bmal1*^IEC−/−^x*IL-10*^−/−BL6^ mice, whereas intermediate phenotypes were found in *IL-10*^−/−BL6^ mice (Supplementary Fig. 6D, E). Taken together, these data demonstrate that a lack of the intestinal clock promotes sensitivity to acute and chronic colon inflammation.

### Restricted feeding requires a functional intestinal clock to ameliorate colitis symptoms

To evaluate whether the beneficial effect of RF on the severity of GI inflammation observed in *IL-10*^−/−Sv129^ mice is caused directly by restoration of the gut clock function, *Bmal1*^IEC−/−^x*IL-10*^−/−BL6^ mice, *IL-10*^−/−BL6^ mice and controls were exposed to RF conditions (Fig. 6F). RF in *IL-10*^−/−BL6^ mice restored clock gene expression to control levels at CT13, whereas its expression was not restored in *Bmal1*^IEC−/−^ mice or *Bmal1*^IEC−/−^x*IL-10*^−/−BL6^ mice due to a lack of intestinal *Bmal1* (Fig. 6A, Supplementary Fig. 6F). Assessment of the humane endpoint of the experiment revealed that RF failed to improve the survival rate of *Bmal1*^IEC−/−^x*IL-10*^−/−BL6^ mice (9/15, 60%), but RF significantly improved the survival rate of *IL-10*^−/−BL6^ mice (9/9, 100%) (Fig. 6G). Consistently, according to the pathohistological evaluation, CD4^+^ T-cell recruitment and colonic *Tnf* expression were greater than the control levels in *IL-10*^−/−BL6^ mice under RF conditions (histology: *p* = 0.0138, CD4 cell recruitment: *p* = 0.0069, TNF expression: *p* = 0.0404) but remained indistinguishable between AD and RF conditions in *Bmal1*^IEC−/−^x*IL-10*^−/−BL6^ mice (histology: *p* = 0.7718, CD4 + T-cell recruitment: *P* = 0.6826, TNF expression: *P* = 0.7619; Fig. 6H–K), suggesting that the intestinal clock gates the beneficial effect of RF. Notably, RF in *Bmal1*^IEC−/−^x*IL-10*^−/−BL6^ mice enhanced species richness to control levels, whereas the microbiota remained arrhythmic (Supplementary Fig. 6G), indicating that a loss of microbial rhythms might be involved in the disease phenotype rather than the overall amount of species. Indeed, we identified 40 zOTUs that gained rhythmicity during RF in *IL-10*^−/−BL6^ mice but remained arrhythmic in *Bmal1*^IEC−/−^x*IL-10*^−/−BL6^ mice. The majority of these zOTUs belonged to the intestinal clock-controlled [11] families *Lachnospiraceae, Muribaculaceae* and *Oscillospiraceae* (Supplementary Fig. 6H, Supplementary Table 5).

In summary, these results clearly demonstrated that intestinal clock dysfunction significantly contributes to colitis severity and that RF improved GI inflammation by targeting intestinal clock functions. Moreover, we provide the first evidence that the intestinal clock gates the inflammatory response and can be directly targeted by RF to mediate the severity and progression of IBD symptoms.

## Discussion

Alterations in clock genes were found in colonic biopsy samples from IBD patients [12]. In this study on *IL-10*^−/−^ mice, a previously described IBD-relevant mouse model of colitis [16], we provide evidence that a disrupted colonic circadian clock is a cause rather than a consequence of GI inflammation in IBD. We demonstrated that specific loss of the circadian function of the IEC clock enhances the severity and prevalence of DSS-induced colitis symptoms in mice. This finding is in accordance with previous literature showing that a lack of colonic *Bmal1* worsened DSS-induced colitis [30].

In a novel genetic mouse model of colitis that also lacks a functional intestinal clock, we provide the first evidence for a cause‒effect relationship between intestinal clock function and colitis. These *Bmal1*^IEC−/−^x*IL-10*^−/−BL6^ mice develop a severely enhanced immune phenotype and experience dramatically reduced survival rates.

Potential mechanisms by which a disrupted colon clock in, e.g., *IL-10*^−/−^ mice, might affect GI inflammation include genes involved in local immune functions and epithelial–microbe interactions. The intestinal clock regulates important inflammatory marker genes, such as *Tnf*, which are arrhythmic and enhanced in *IL-10*^−/−^ mice and have previously been linked to the development of IBD [32].

Intestinal clock-controlled genes are also involved in immune cell recruitment, such as *Ffar2*, which plays an important role in intestinal homeostasis by regulating immune cell abundance in colonic lymphoid tissues and the secretion of mucus-associated proteins and antimicrobial peptides [24], as well as *Abcc2*, which actively regulates mucosal inflammation [26]. Both genes lost rhythmicity in *Bmal1*^IEC−/−^ mice. Moreover, *Rorc*, which encodes the IBD risk factor RORγ [25], was elevated and arrhythmic in mice lacking an intestinal clock. These results further suggest the importance of the intestinal clock in modulating the IBD-like phenotype.

In addition, the expression of the promigratory genes *Vcam-1* and *Cxcr4* undergoes diurnal oscillation in multiple lymphoid and nonlymphoid tissues [28, 29]. Here, we revealed that the circadian rhythm of the expression of these two genes in the colon requires a functional intestinal clock. For example, the expression of *Vcam-1* and *Cxcr4* was arrhythmic in colonic tissues from mice harboring a dysfunctional intestinal clock (*IL-10*^−/−Sv129^ and *Bmal1*^IEC−/−^). Furthermore, *Cxcr4* mediates diurnal oscillations in T-cell distribution and responses [28], and *Vcam-1* is involved in the rhythmic pattern of leukocyte migration [29]. Therefore, the arrhythmicity of these genes likely contributes to immune cell recruitment to the colon LP and thus impacts the local immune response.

Here, we provide the first evidence for a functional role of the intestinal clock in regulating rhythmic immune and inflammatory processes, including immune cell recruitment and the microbiota composition, which are both key elements in IBD development and progression [32]. Dysfunctional colon clocks in *IL-10*^−/−^ and *Bmal1*^IEC−/−^ mice caused a loss of rhythmicity in the recruitment of T cells to the colonic lamina propria. Similarly, rhythmic leukocyte trafficking into the bloodstream and infiltration to the lymph nodes, spleen and bone marrow have previously been described for T cells and monocytes and depend on cell- and tissue-specific clocks [33].

The physiological importance of diurnal immune cell recruitment was demonstrated in lymphoid organs, which improved the immune response to antigens and bacterial infection [28]. Specific clustering of intestinal CD4+ cells induced colitis in an adoptive transfer mouse model [34]. Consequently, arrhythmic leukocyte recruitment to the colon might augment the inflammatory response in mice with a disrupted colon circadian clock.

The microbiota composition and function are associated with the development of GI inflammation [35], and the arrhythmicity of the microbiome is linked to the development of obesity and type 2 diabetes [36]. Interestingly, microbiome rhythmicity was disrupted in *IL-10*^−/−Sv129^ mice. This was likely caused by intestinal clock dysfunction in these mice because the intestinal clock was recently identified as a major driver of microbiota rhythmicity [11]. Indeed, *Bmal1*^IEC−/−^x*IL-10*^−/−BL6^ mice with a genetically dysfunctional intestinal clock exhibit a more pronounced loss of microbial rhythmicity than *IL-10*^−/−BL6^ mice. Similar to previous results obtained from *Bmal1*^IEC−/−^ mice [11], intestinal clock-controlled taxa involved in SCFA fermentation and secondary bile acid formation lost rhythmicity in *IL-10*^−/−Sv129^ mice and thereby likely caused arrhythmicity in the levels of specific BAs, such as DCA, 7-sulfo CA, HDCA and α-MCA, which have frequently been linked to intestinal inflammation [37]. Notably, reduced levels of secondary BAs, e.g., DCAs and HDCAs, identified in *Bmal1*^*IEC*−/−^ mice receiving disease-associated microbiota were also found in patients with UC, and supplementation, particularly with DCA, ameliorated experimental colitis in mice [20]. Therefore, the generation of arrhythmic microbial-derived products due to the composition of the arrhythmic microbiota in *IL-10*^−/−^ mice might promote their inflammatory immune response.

Indeed, our recent transfer experiments provided evidence that the arrhythmic microbiota can alter GI immune homeostasis [11]. However, the transfer of the arrhythmic microbiota from *Bmal1*^*IEC*−/−^ did not increase the severity of inflammation in germ-free *IL-10*^−/−^ recipients (although 15% more IL-10^−/−^ mice developed inflammation), suggesting that intestinal clock dysfunction rather than the loss of microbial rhythmicity induces colonic inflammation. Indeed, the transfer of IBD-associated microbiota provides direct evidence for the physiological relevance of the intestinal circadian clock in the microbiota-induced inflammatory response. In particular, the findings in *Bmal1*^*IEC*−/−^ recipients reflect the immune response and altered microbiome observed in *IL-10*^−/−^ donors and thus indicate an activated, early inflammatory response, although in colon cross-sections, no pathohistological tissue changes could be observed between genotypes. This might be due to patchy colonic inflammation associated with the Crohn’s disease-like phenotype reported in *IL-10*^−/−^ mice [38]. Future transfer experiments (long-term and short-term) in *IL-10*^−/−^ mice using “Swiss rolls” to investigate inflammation in the entire intestine might clarify whether the loss of microbial rhythmicity per se rather than the IBD-associated microbiota can promote gastrointestinal inflammation.

Taken together, these results highlight the relevance of intestinal clock function for GI inflammatory processes involved in microbiota-induced IBD development. Interestingly, a functional intestinal clock in control recipients was capable of restoring the circadian time differences of zOTUs. These rhythmic hosts showed reduced immune cell recruitment into the LP and inflammatory marker gene expression, indicating that the restoration of intestinal clock functions might reduce GI inflammation.

To determine whether intestinal clock function can be targeted by meal timing to influence the immune response to GI inflammation, *IL-10*^−/−^ mice were exposed to nighttime-restricted feeding (RF). Here, we demonstrated that rhythmicity and the amplitude of the colonic circadian clock can indeed be restored by RF in a diseased mouse model (*IL-10*^−/−^) with disrupted colonic clock function. Moreover, targeting the colon clock by RF further led to the restoration of rhythmic colon clock functions, such as CD4^+^ T-cell recruitment into the colonic LP and microbiome oscillations. This finding is in line with the literature showing that RF in rodents influences various peripheral clocks [9, 15] and genes involved in inflammatory signaling, such as NF-kB, TLR and IL-17, in many tissues, including the intestine [39]. Consistent with our hypothesis, RF further ameliorated the IBD-like colitis phenotype in *IL-10*^−/−^ mice and substantially enhanced their survival rate. This finding is in accordance with results obtained in mice showing that RF protects against DSS-induced colitis by affecting intestinal functions [40]. Interestingly, meal timing in humans has been tested for the treatment of a variety of diseases, including diabetes, cardiovascular diseases and cancer [13], and inconsistent meal times could be correlated with IBD symptoms [41], indicating that RF might become a strategy for the treatment of IBD patients. However, further experiments are required to validate the long-term effect of RF.

Importantly, we provide the first direct mechanistic evidence that the effect of RF on the severity of IBD is directly gated by the intestinal clock because RF fails to reduce the inflammatory phenotype (immune cell infiltration, inflammatory marker gene expression, histopathological scores) when the intestinal clock is genetically dysfunctional in *Bmal1*^*IEC*−/−^x*IL-10*^−/−^ mice. Our results clearly demonstrated that intestinal clock dysfunction significantly contributes to colitis severity and that RF improved GI inflammation by targeting intestinal clock functions. Notably, we observed improved microbiota richness following RF in *Bmal1*^*IEC*−/−^x*IL-10*^−/−^ mice (Supplementary Fig. 6B, G), which has been associated with improved IBD conditions [42]. Thus, we cannot exclude the possibility that other factors, such as the microbiome, which is known to respond to RF [8] independent of the intestinal clock, might also contribute to improved disease outcomes. Nevertheless, no significant improvements in tissue pathology, the inflammatory response or overall survival following RF were observed in *Bmal1*^*IEC*−/−^x*IL-10*^−/−^ mice. Thus, these results demonstrate that the intestinal clock represents a target for the treatment of GI inflammation. Whether the beneficial effect of RF is solely due to intestinal clock functions or can be affected by additional factors remains to be addressed in future experiments. For example, the role of the intestinal clock in mediating the effect of RF could be further validated by subjecting intestinal clock-deficient mice to DSS treatment and placing them under RF or AD-fed conditions.

Taken together, our results demonstrate that a functional intestinal clock is essential for maintaining GI homeostasis and is a major player in IBD progression. In addition, our findings suggest that enhancing intestinal clock function by meal timing might become the next step in developing novel strategies for circadian therapies for IBD and potentially other metabolic diseases in humans.

## Methods

### Ethics statement and mouse models

For details, see the supplementary material.

### Behavior analysis

Handling and activity measurements during the experiments were performed as previously described [43]. Wheel-running activity was analyzed using ClockLab software (Actimetrics). The last 10–14 days of each condition were used to determine the period (tau, calculated using an X^2^ periodogram and confirmed by fitting a line to the onset of activity), the duration of the active period (alpha), the amount of activity, and the subjective day/night activity ratio (where the subjective day under DD conditions is the inactive period between the cessation of activity and the onset of activity and the subjective night is the active period between the onset of activity and the cessation of activity). The average daily food intake was measured in the second week under different light conditions and *ad libitum* feeding conditions. For the restricted feeding condition, the average daily food intake was measured manually over 3 consecutive days in the last week of the restricted feeding period.

### Light–dark (LD) and constant darkness (DD) conditions

Male *IL-10*^−/−Sv129^ mice and their wild-type littermates were housed individually at 8 weeks of age under an LD cycle for 2 weeks (aged 8–10 weeks), switched to a DD cycle for 2 more weeks (aged 10–12 weeks) and subsequently returned to the LD phase.

### Nighttime-restricted feeding

Fourteen-week-old *IL-10*^−/−Sv129^ mice and wild-type littermates were gradually introduced to the restricted feeding (RF) protocol starting with 8 h of food availability (ZT13-ZT21) for the first 3 days, followed by 6 h of food availability (ZT13-ZT19) for the following 3 days and finally 4 h of food availability (ZT13-ZT17) for four weeks. The same RF regimen was also applied to *Bmal1*^flox/flox^, *Bmal1*^IEC−/−^, *IL-10*^−/−BL6^ and *IL-10*^−/−BL6^ x *Bmal1*^IEC−/−^ mice, with an earlier start point at the age of 10 weeks. Tissues were harvested at the end of the 4 week RF period on the 2nd day in constant darkness.

### Tissue collection

For details, see the supplementary material.

### Organoids

For details, see the supplementary material.

### Gene expression analysis

For details, see the supplementary material.

### RNA sequencing

RNA quality was verified using an Agilent2100 Bioanalyzer with RNA 6000Nano Reagents. Library preparation and rRNA depletion were performed using the TruSeq Stranded mRNA Library Prep Kit. After the final QC step, the libraries were sequenced in a paired-end mode (2 × 150 bases) on a Novaseq6000 sequencer (Illumina) with a depth of ≥ 12 million paired reads per sample.

### Pre-processing

The quality of the next-generation sequencing data was assessed with FastQC v0.11.5 (RRID:SCR_014583, http://www.bioinformatics.babraham.ac.uk/projects/fastqc/), and low-quality samples were filtered out. Adapter content and low-quality reads were removed using Trimmomatic v0.39 [44].

Trimmed FASTQ files were then mapped against the mouse mm10 genome with the STAR v2.7.5c aligner [45]. Format conversions were performed using SAMtools v1.3.1 [46]. The featureCounts program v1.4.6 [47] was used to count reads located within an exon that did not overlap with multiple features, with a threshold of MAPQ ≥ 4, and that were not chimeric.

### Normalization and differentially expressed gene analysis

DESeq2 version 1.22.0 (RRID:SCR_015687 [48]) was used to normalize the read count matrix and perform differential expression analysis. The Bioconductor package “biomaRt” version 2.38 (RRID:SCR_002987 [49]) was used to map the MGI symbols to Ensembl gene IDs. To identify general DEGs between WT and KO mice, an initial model (Genotype + Time) with a filtering FC > 1.5 and Benjamini‒Hochberg-Adj.*p* < 0.05 was used to identify the differentially expressed genes (DEGs). Then, a multimodel (Genotype + Period + Genotype: Period) was used to compare day vs. night differences.

Gene Ontology biological process enrichment was performed using clusterProfiler (RRID: SCR_016884) [50], and GO terms were considered to be significantly enriched when the *q* value was <0.05. All genes expressed with a minimum of 1 count in any of the samples were used as the background. Redundant terms were removed manually.

### Circadian analysis

The rhythmicity of the oscillating transcripts was measured by the JTK cycle [51] through the MetaCycle R package [52]. With the settings of Period=24 h and adj.*p* < 0.05, filtered genes were then defined as rhythmic. The package compareRhythms [53] was modified to compare rhythmicity differences in transcripts between control and *Bmal1*^IEC−/−^ mice as previously described [11].

The associated RNA-seq data are available at the National Center for Biotechnology Information (NCBI) Gene Expression Omnibus (GEO), https://www.ncbi.nlm.nih.gov/geo/query/acc.cgi?acc=GSE230078.

### High-throughput 16S ribosomal RNA (rRNA) gene amplicon sequencing analysis

For details, see the supplementary material.

### Targeted and untargeted metabolite analyses

For details, see the supplementary material. The associated untargeted metabolomics data are available at MSV000091092.

### Dextran sulfate sodium (DSS)-induced acute experimental colitis

Fourteen-week-old male *Bmal1*^*IEC*−/−^ mice and controls were maintained in constant darkness after the experimental protocol started. Briefly, *Bmal1*^*IEC*−/−^ and control mice were administered 2% DSS dissolved in drinking water for 5 days, followed by normal water for 2 days. The onset of disease in each mouse was calculated separately, and the mice were sacrificed at circadian time (CT) 7 on day 7, when most of the immune cells peaked in the colon lamina propria.

### Transfer experiment

For details, see the supplementary material.

### Immune cell isolation from the lamina propria

For details, see the supplementary material.

### Flow cytometry measurement

For details, see the supplementary material.

### Histology

For details, see the supplementary material.

### PICRUST 2.0

The sequences of the zOTUs that exhibited rhythmicity after restricted feeding in the *IL-10*^−^^/−Sv129^ group were used to construct the metagenome using PICRUST2.0 [54] for the prediction of metagenomic functionality. The corrected zOTU 16 S rRNA gene copy number was multiplied by the predicted functionality to predict the metagenome. The resulting enzymatic genes classified according to the Enzyme Commission (EC) numbers were mapped to Metacyc pathways. Superclasses were removed, and the metabolic pathway abundance was used for statistical analysis using STAMP (2.1.3). Statistical differences were calculated based on White’s nonparametric *t* test and the Benjamini–Hochberg–Dales discovery rate to adjust for multiple testing.

### Statistical analyses

Statistical analyses were performed using GraphPad Prism, version 9.0.0 (GraphPad Software), JTK_cycle v3.1. R [51] or R. Between-sample microbiota diversity was calculated by generalized UniFrac using GUniFrac v1.1. distances within the Rhea [55] pipeline. Microbiota composition comparison was calculated by the PERMANOVA test on a generalized UniFrac dissimilarity matrix and illustrated by MDS [55]. Circadian profile graphs and phase calculations were analyzed by fitting a cosine-wave equation: *y* = baseline + (amplitude∙cos(2 ∙ π  ∙  ((x-[phase shift)/24]))). The nonparametric algorithm JTK_cycle was used for overall rhythmicity of all zOTUs and transcripts. Connected curves in the circadian profile graphs within the figures indicate significant rhythmicity based on cosine analyses, whereas connected straight lines indicate nonsignificant cosine fits. Comparison of rhythms between datasets was performed by the adjusted version of compareRhythms as previously described [11]. The amplitude calculations depicted in the Manhattan plots are based on the output of JTK_cycle, and the phase was calculated by cosine-wave regression. Comparisons between two groups were performed using the nonparametric Mann‒Whitney test (two-sided). Two-way ANOVA followed by Benjamini‒Hochberg correction was used to compare datasets with two categorical variables. Three-way ANOVA following Benjamini–Krieger–Yekutieli correction was used to compare datasets containing three categorical factors. A *p* value ≤ 0.05 was assumed to indicate statistical significance.

## Supplementary information

Supplemental Figure 1

Supplemental Figure 2

Supplemental Figure 3

Supplemental Figure 5

Supplemental Figure 4

Supplemental Figure 6

Supplemental Figure Legends

Supplemental Table 1

Supplemental Table 2

Supplemental Table 3

Supplemental Table 4

Supplemental Table 5

Supplemental Table 6

Supplemental Material
