# Supplementary figures and images for: Targeting the intestinal circadian clock by meal timing ameliorates gastrointestinal inflammation
Source: Cell Mol Immunol. 2024 Jun 25;21(8):842–55. doi: 10.1038/s41423-024-01189-z (PMC11291886; doi:10.1038/s41423-024-01189-z)

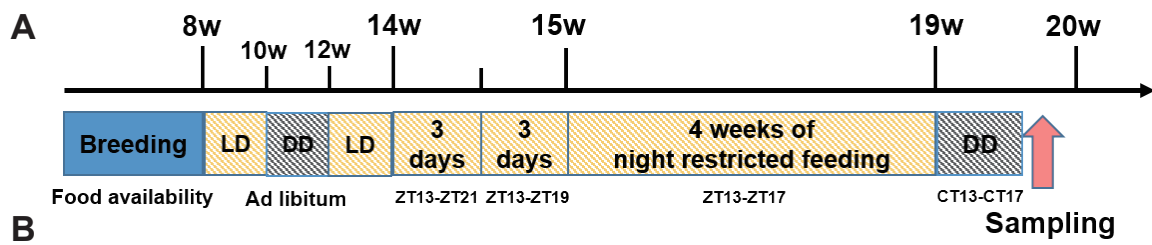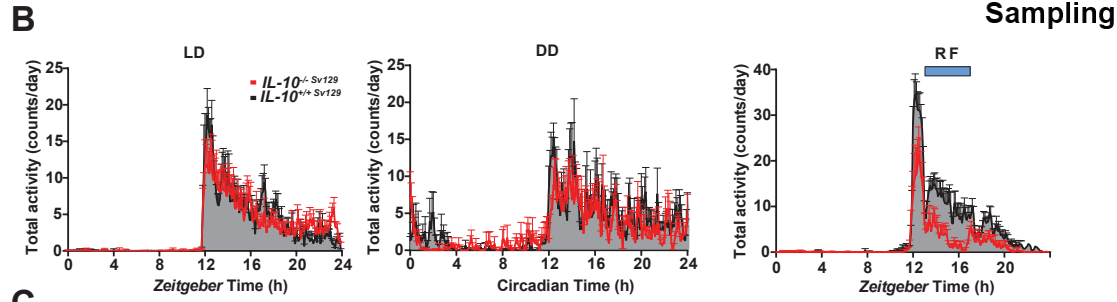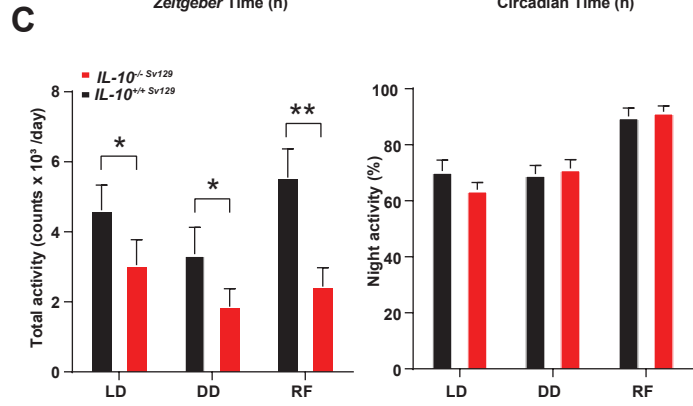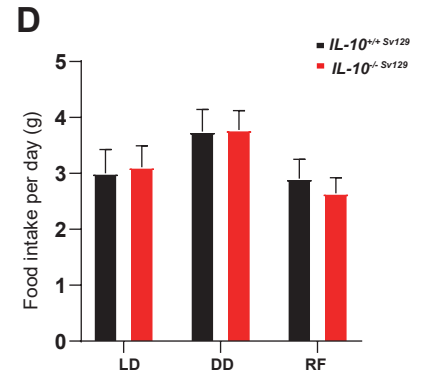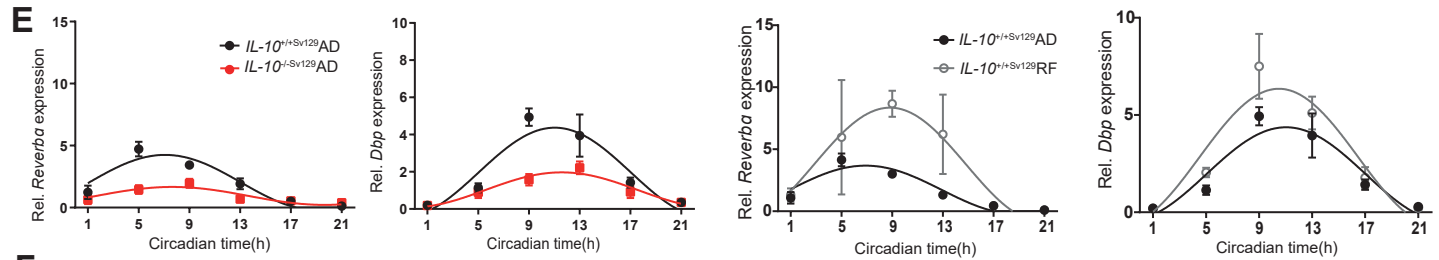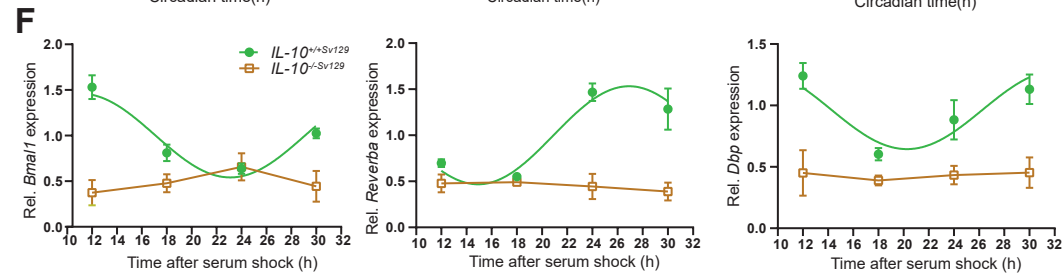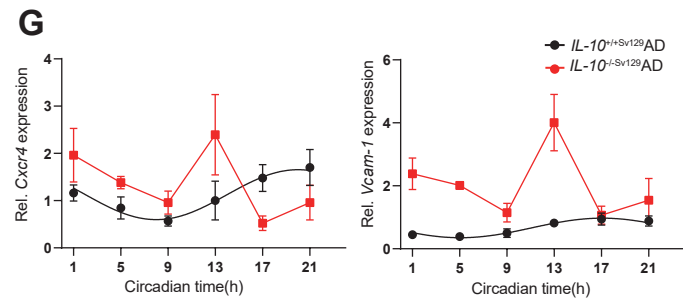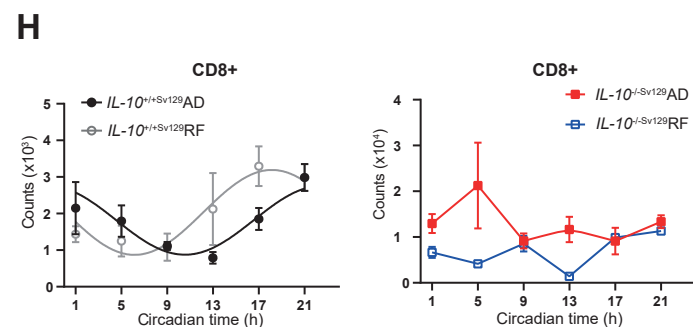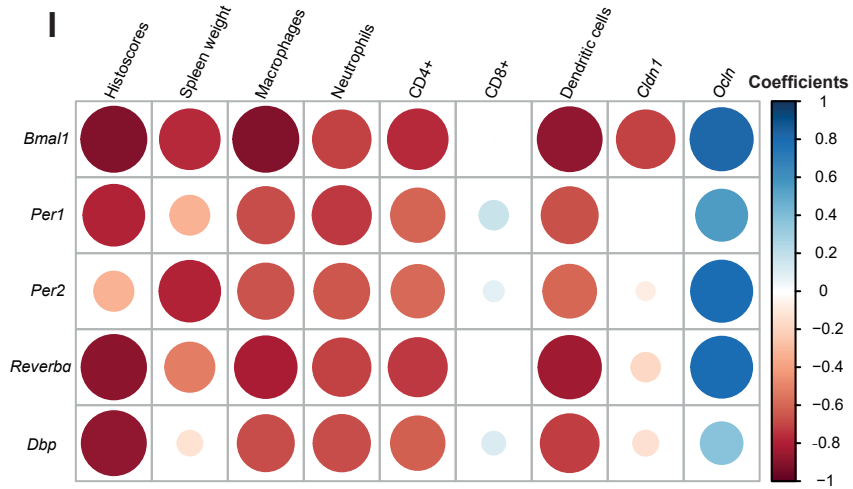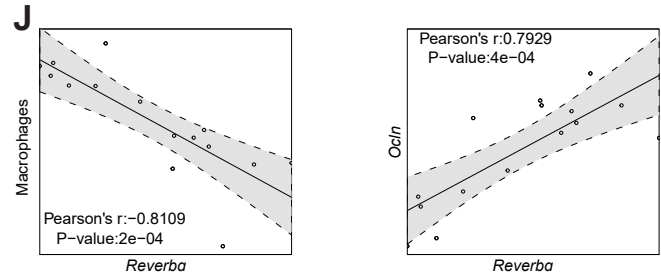

Supplement: Supplementary file 1 — Supplemental Figure 1 [file 41423_2024_1189_MOESM1_ESM.pdf]

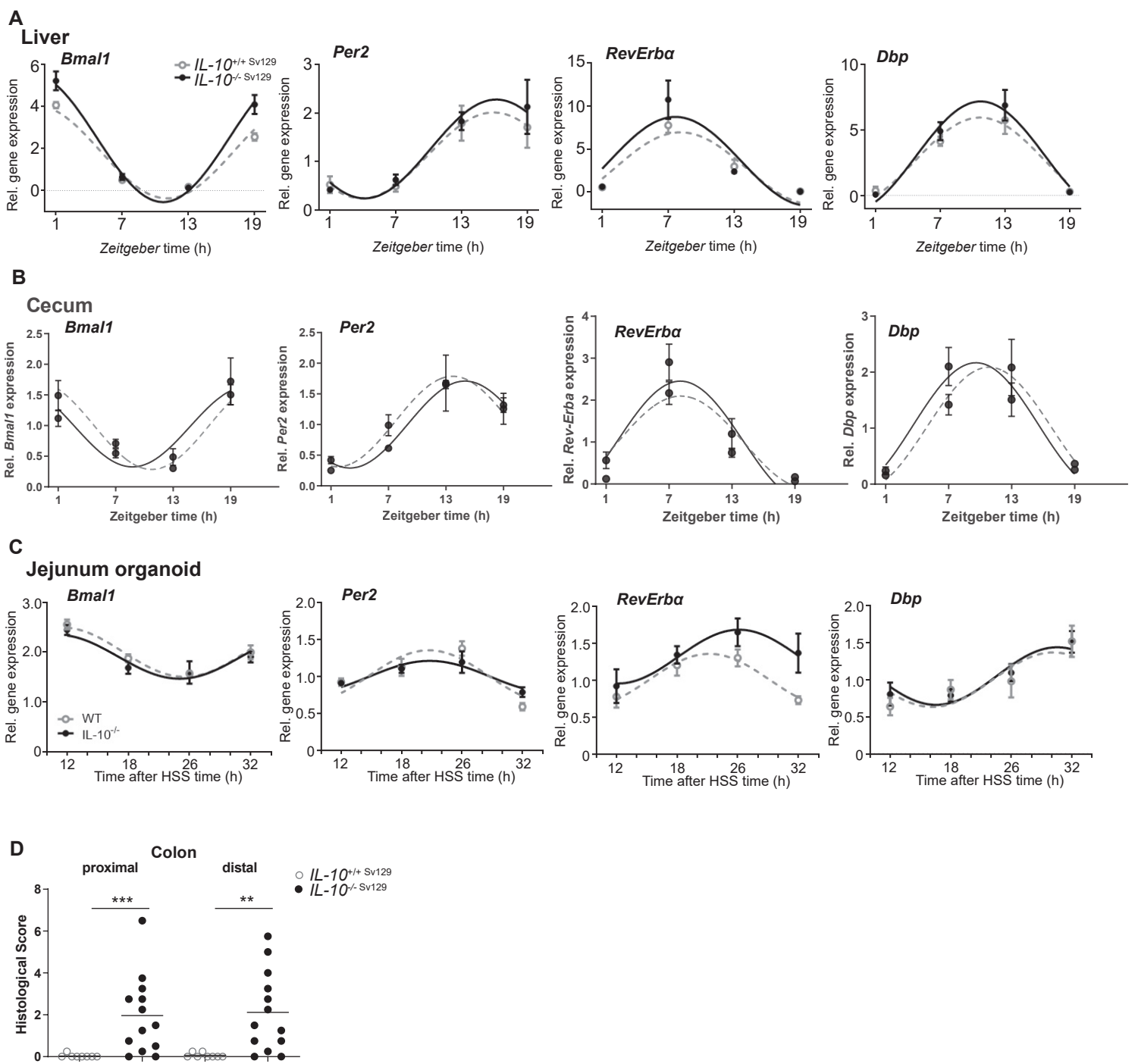

Supplement: Supplementary file 2 — Supplemental Figure 2 [file 41423_2024_1189_MOESM2_ESM.pdf]

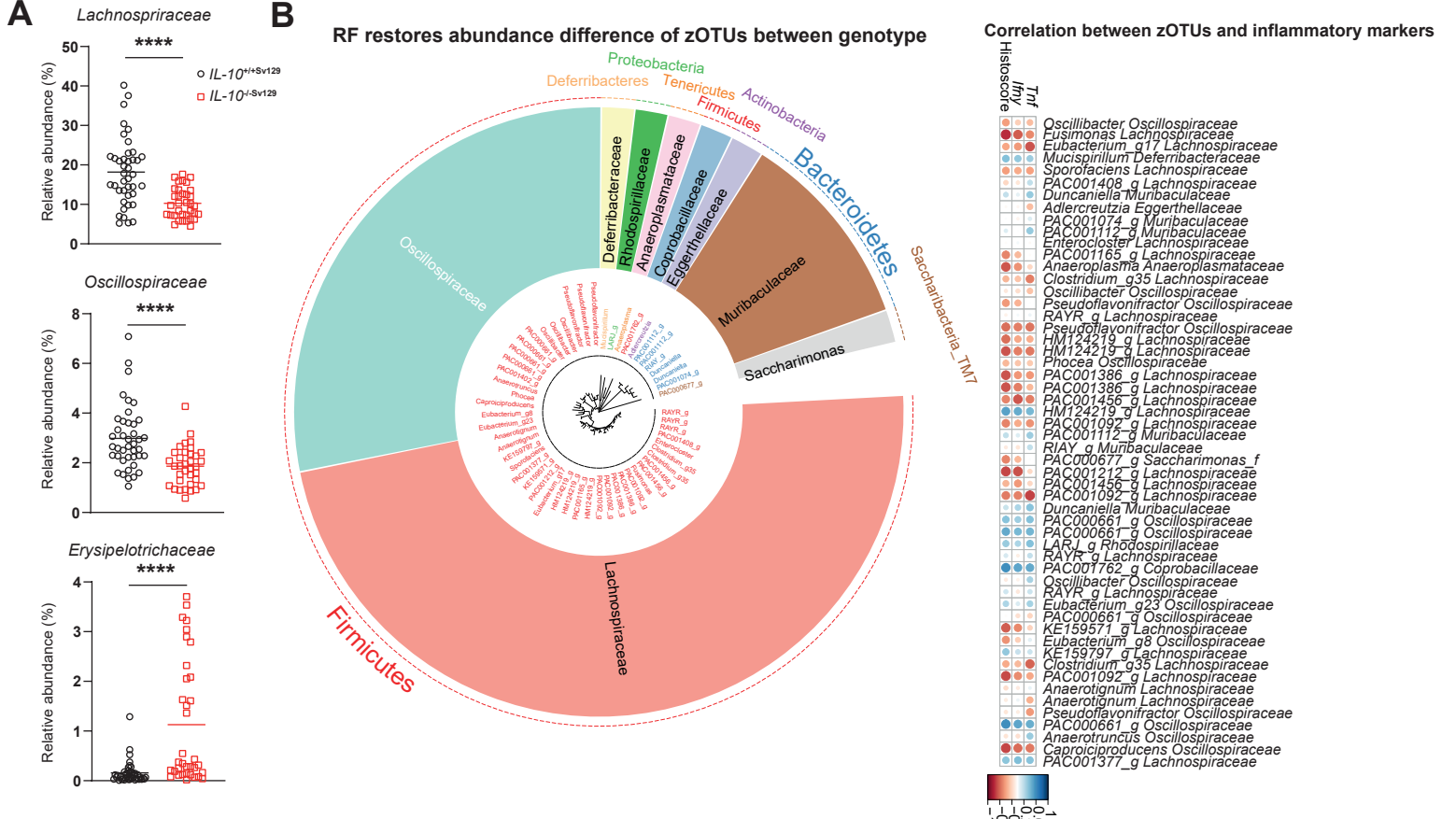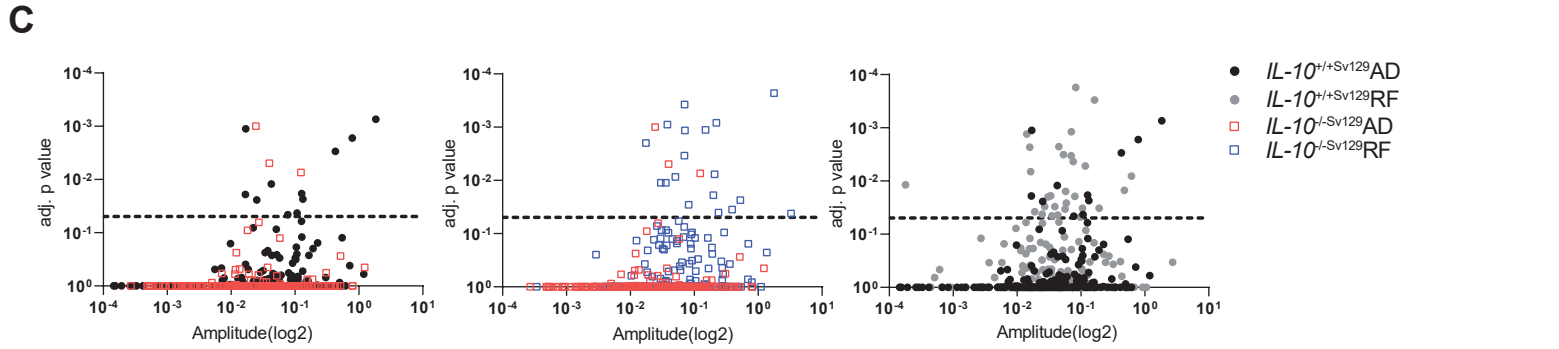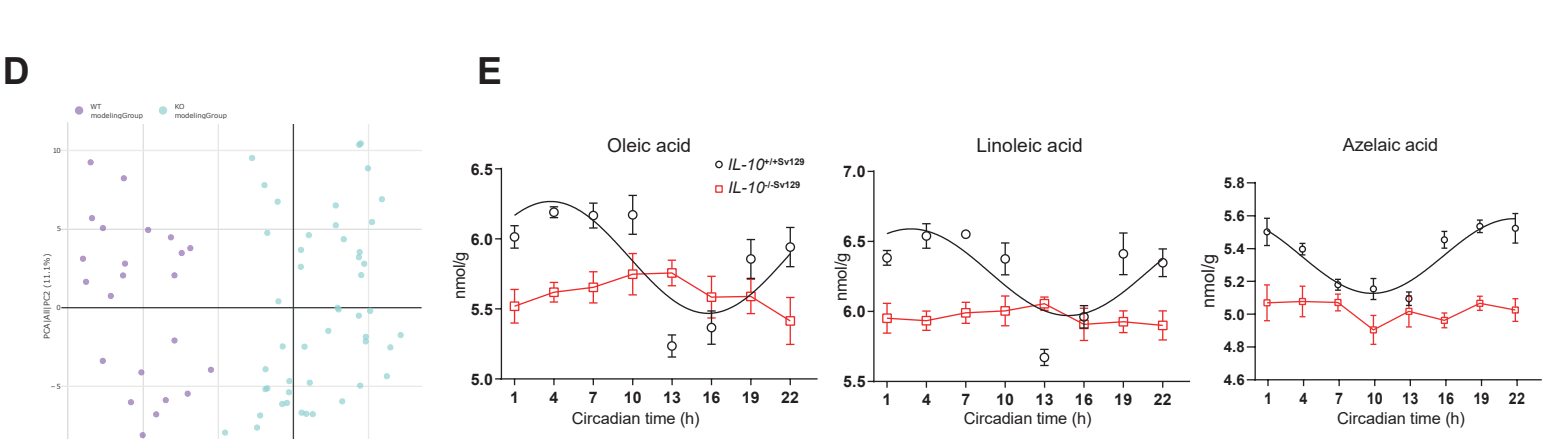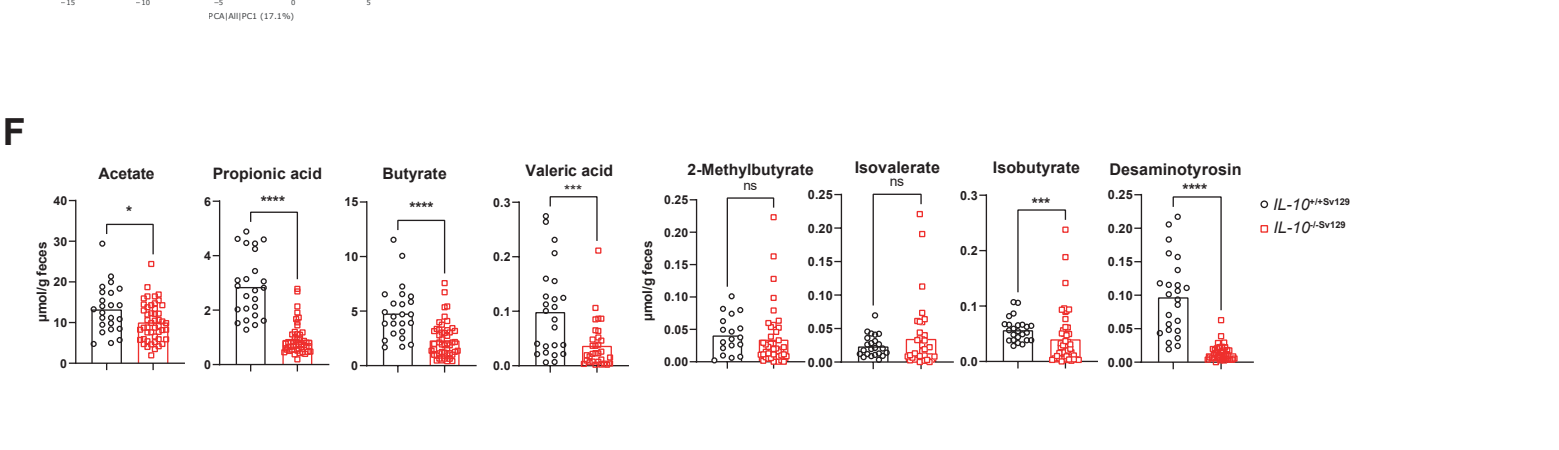

Supplement: Supplementary file 3 — Supplemental Figure 3 [file 41423_2024_1189_MOESM3_ESM.pdf]

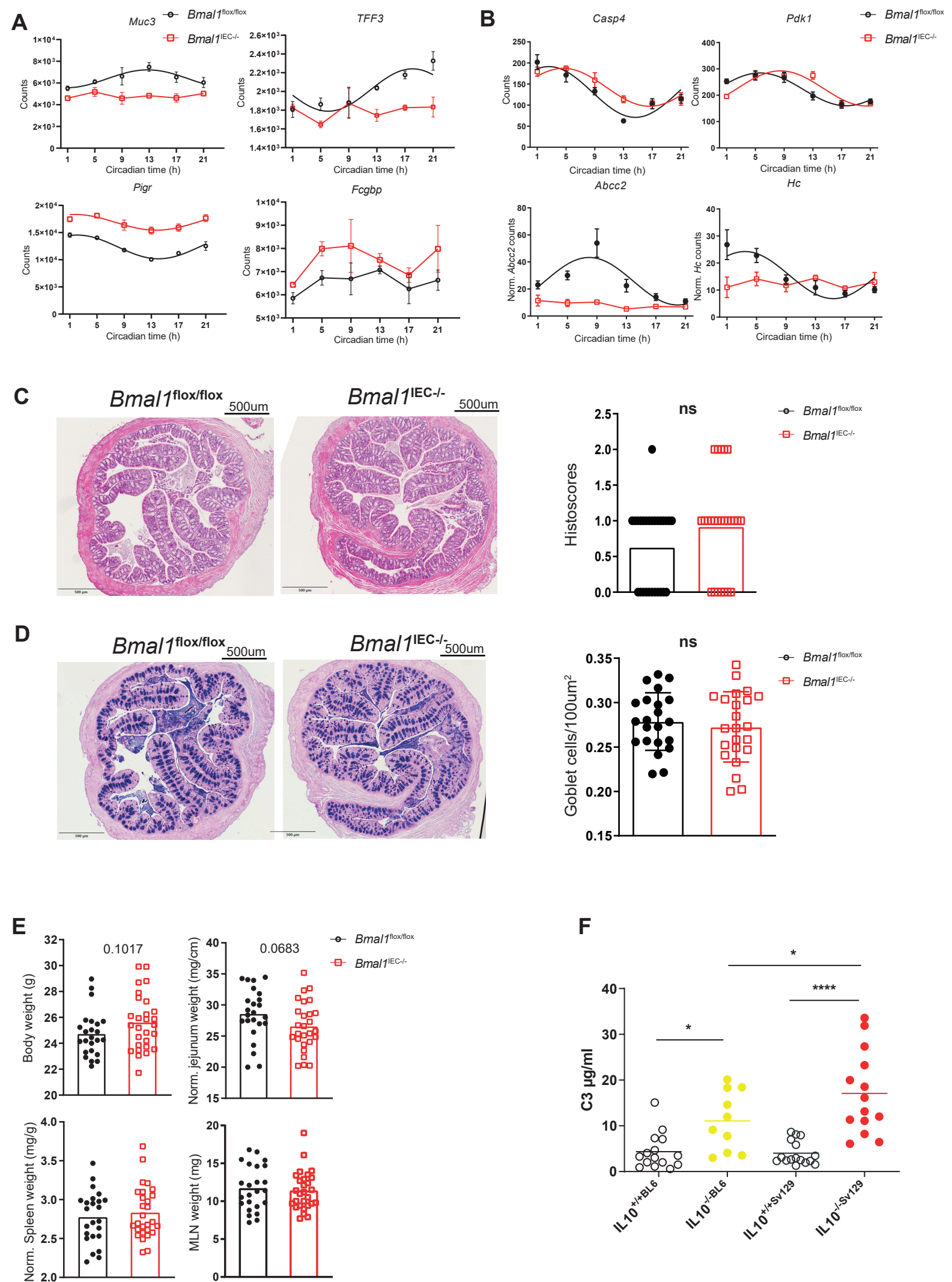

Supplement: Supplementary file 4 — Supplemental Figure 5 [file 41423_2024_1189_MOESM4_ESM.pdf]

**A**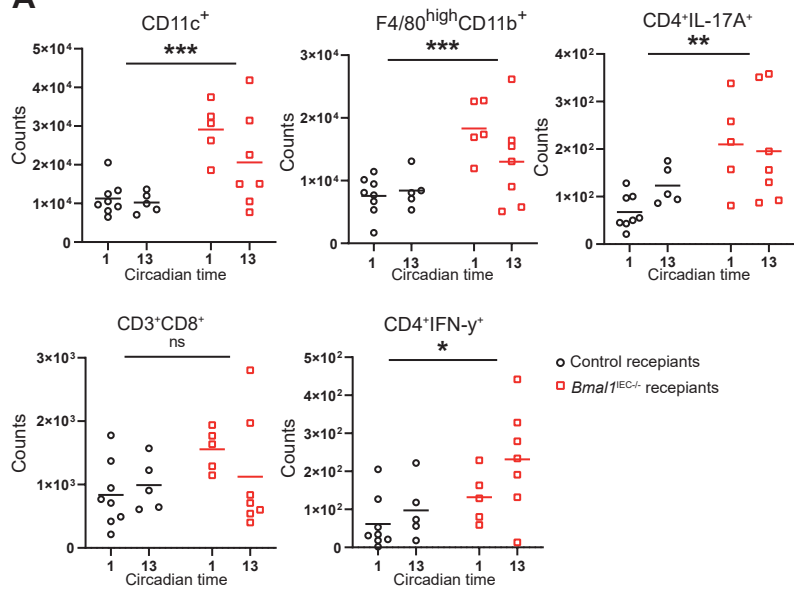**B**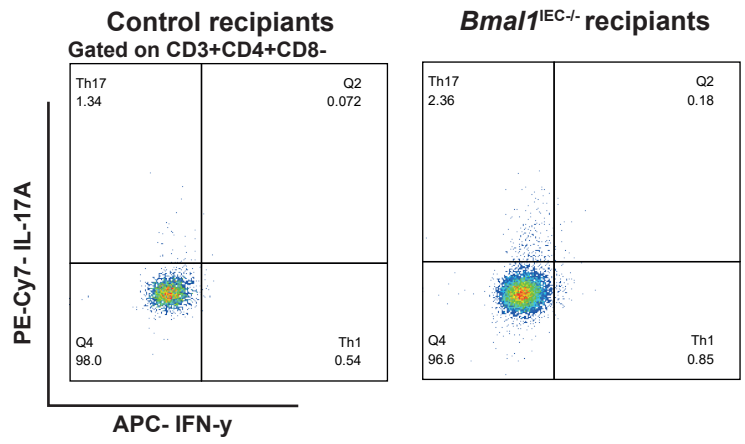**C**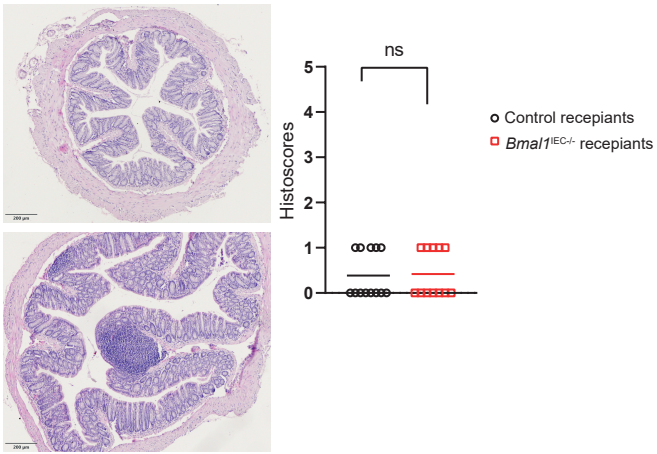**D**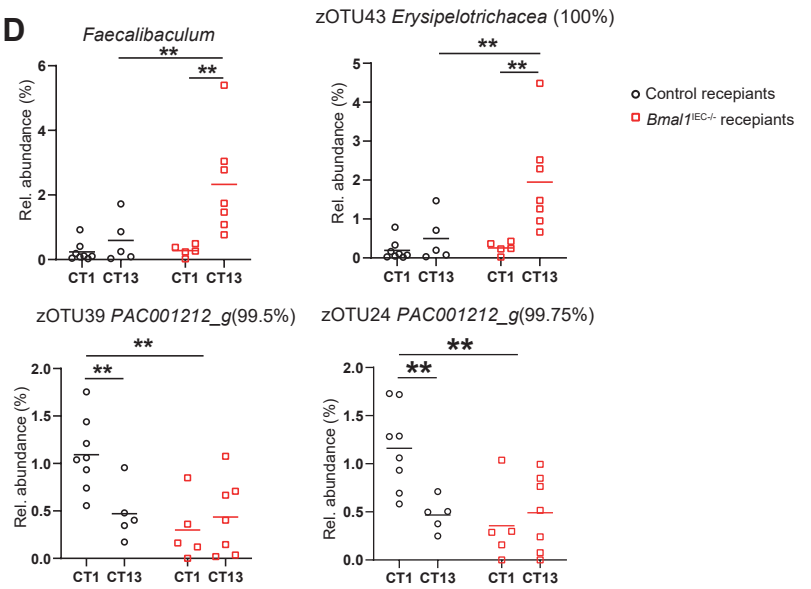

Supplement: Supplementary file 5 — Supplemental Figure 4 [file 41423_2024_1189_MOESM5_ESM.pdf]

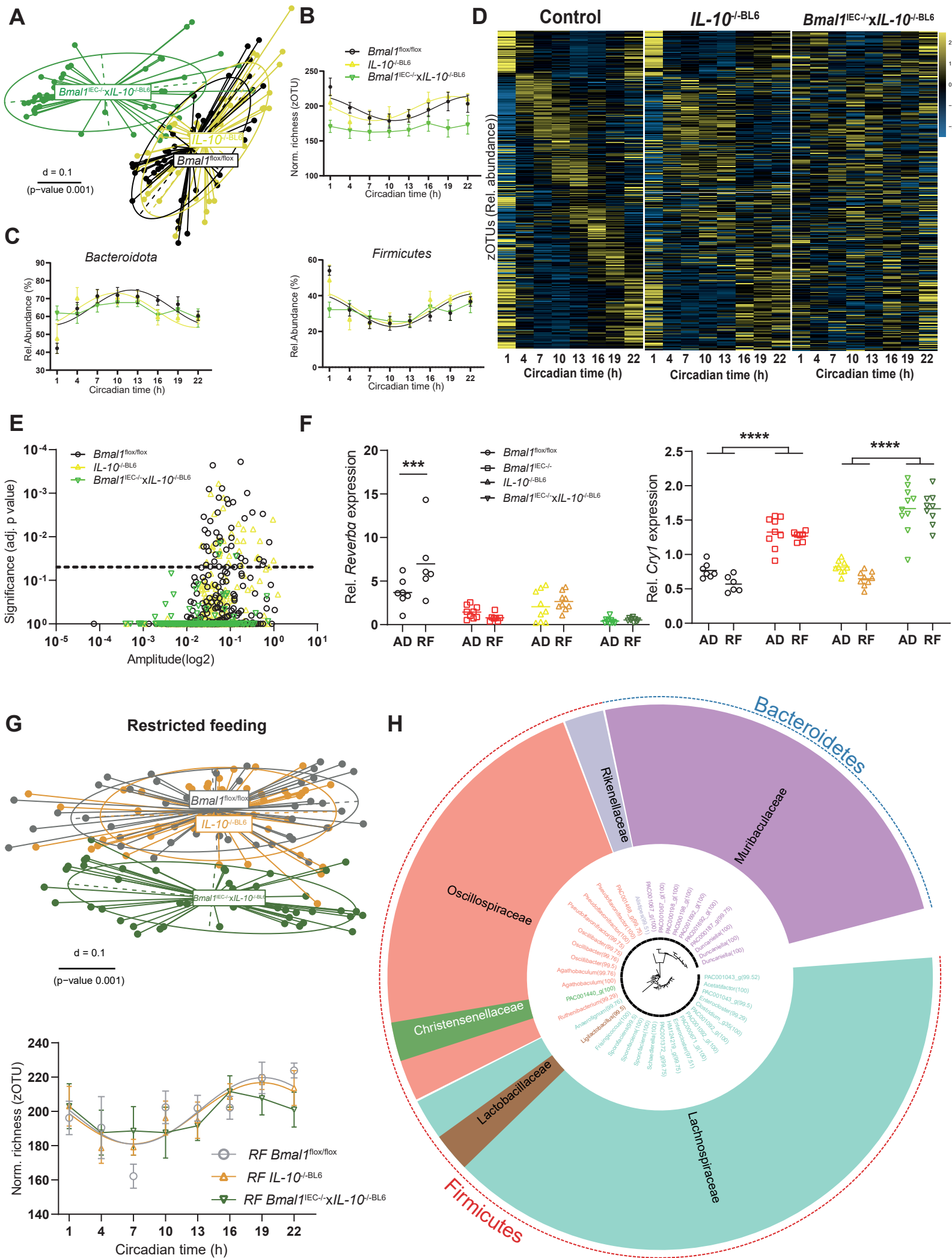

Supplement: Supplementary file 6 — Supplemental Figure 6 [file 41423_2024_1189_MOESM6_ESM.pdf]
