# Supplementary material for: Targeting the intestinal circadian clock by meal timing ameliorates gastrointestinal inflammation
Source: Cell Mol Immunol. 2024 Jun 25;21(8):842–55. doi: 10.1038/s41423-024-01189-z (PMC11291886; doi:10.1038/s41423-024-01189-z)
Supplement: Supplementary file 7 — Supplemental Figure Legends [file 41423_2024_1189_MOESM7_ESM.docx]

**SUPPLEMENTAL LEGENDS**

*Supplement Figure 1 Circadian phenotype of IL-10^-/-Sv129^mice*

(A) Schematic illustration of the experimental plan. (B) Representative graph of total activity of control and *IL-10^-/-^*^SV129^ mice for an individual day and (C) Quantification of total activity. (D) Food intake of control and *IL-10^-/-^*^SV129^ mice in LD, DD and RF. Clock genes expression in (E) colon tissues and (F) colonic organoids from control and *IL-10^-/-^*^SV129^ mice. (G) Circadian profile of relative gene expression of *Cxcr4* and *Vcam-1* in colonic tissues from control and *IL-10*^-/-Sv129^ mice. (H) Circadian profile of the amount of CD8+ cells in colon lamina propria from control and *IL-10^-/-^*^SV129^ mice under AD and RF conditions. (H) Graphical display of all variables combinations in a matrix. Each correlation is depicted as a circle coloured according to the direction of correlation coefficients (negative, red; positive, blue). The size of the circles is dictated by the uncorrected p-value. (J) Representative correlation plot from (I). n>3/time points for each group. Significant rhythms are illustrated with fitted cosine-regression; data points connected by straight lines indicate no significant cosine fit curves (p > 0.05) and thus no rhythmicity. Significance were calculated by two-way ANOVA following Benjamini-Hochberg correction, * p ≤ 0.05, ** p ≤ 0.01, *** p ≤ 0.001, **** p ≤ 0.0001. Data are represented as mean ± SEM.

*Supplement Figure 2 Clock genes expression of liver, cecum and jejunum organoid of IL-10^-/-Sv129^ mice and controls*

Circadian clock gene expression in peripheral tissues and organoids from 18 weeks old *IL-10*^-/-Sv129^ mice and controls. Rhythmic clock genes expression of liver (A), cecum (B) and jejunal organoids (C) from control and *IL-10*^-/-Sv129^ mice. n=4 mice for each time point in both groups. Significant rhythms are illustrated with fitted cosine-regression or fitted harmonic-regression; data points connected by dotted lines indicate no significant cosine fit curves (p-value > 0.05) and thus no rhythmicity. *IL-10*^-/-Sv129^ (black) and control (dashed grey). (D) Histological score of 18 weeks old IL-10^-/-Sv129^ mice and controls. Data are represented as mean ± SEM. Significance: p-value ≤ 0.05.

*Supplement Figure 3 Alterations in the microbiome between IL-10^-/-Sv129^ mice and controls*

(A) Representative taxa (*Lachnospriraceae, Oscillospiraceae and Erysipelotrichaceae*) which differentially abundant in fecal samples from *IL-10*^-/-Sv129^ mice. (B) Taxonomic tree of fecal microbiota which differentially abundant in *IL-10*^-/-SV129^ mice and reversed after RF. Taxonomic ranks are from phylum (outer dashed ring), family (inner ring highlighted) to genera (middle, color coded according to phylum) which are indicated by the individual branches (left) and correlations of zOTUs in (B) with histoscores, *Tnf* and *Ifn-y* gene expression (right). (C) Manhattan plot of the amplitude and adj.p value of zOTUs identified in control and *IL-10*^-/-Sv129^ mice under AD and RF conditions. (D) PCA-plot of fecal metabolites obtained from control and *IL-10*^-/-Sv129^ mice through untargeted metabolomics and (E) examples of metabolites which lost rhythmicity in *IL-10*^-/-Sv129^ mice. (F) Quantification of SCFA and desaminotyrosine measured by targeted metabolomics in fecal samples from control and *IL-10*^-/-Sv129^ mice. Data points represent individual mice (n>3/time points for each genotype). Significant rhythms are illustrated with fitted cosine-regression; data points connected by straight lines indicate no significant cosine fit curves (p > 0.05) and thus no rhythmicity. Data are represented as mean ± SEM. Statistics were performed by Mann–Whitney U test. Asterisks indicate significant differences *p<0.05, **p<0.01, ***p<0.001.

*Supplement Figure 4 Disease-associated microbiota induce immune responses and microbial alterations in Germ-free Bmal1^IEC-/-^ mice*

(A) Additional immune cells in the colon lamina propria from the recipients and (B) gating strategy for Th1 and Th17 cells. (C) Representative H&E staining scans of colon sections from *Bmal1^IEC-/-^* mice received control and disease-associated microbiota (left) and histopathological scores (right). (D) Representative microbial alteration in genus (*Faecalibaculum*) and specific zOTUs leves. Data points represent individual mice (n>5/time points for each genotype). Significances were calculated by two-way ANOVA following Benjamini-Hochberg correction, asterisks indicate significant differences *p<0.05, **p<0.01, ***p<0.001.

*Supplement Figure 5 Inflammatory phenotype in the colon of Bmal1^IEC-/-^ mice*

(A) Circadian profile of transcripts *Muc3, Tff3, Pig*r and *Fcgbp* and (B) *Casp4, Pdk1, Cxcr4* and *Vcam-1* in control and *Bmal1*^IEC-/-^ mice. (C) Representative scans of H&E staining of colon cross sections from control and *Bmal1*^IEC-/-^ mice (left) and histopathological scores (right). (D) Representative scans of PAS-AB staining of the same colon cross sections with (B) from control and *Bmal1*^IEC-/-^ mice (left) and quantification of goblet cells (right). (E) Body weight, organ weight of jejunum, spleen and MLN of control and *Bmal1*^IEC-/-^ mice. (E) Level of complement 3 in fecal samples from control and *IL-10*^-/-^ mice with BL6 and Sv129 background. Significant rhythms are illustrated with fitted cosine-regression; data points connected by straight lines indicate no significant cosine fit curves (p > 0.05) and thus no rhythmicity. Data points represent individual mice (n=4/time points for each genotype). Data are represented as mean ± SEM. Statistics were performed by Mann–Whitney U test and two-way ANOVA following Benjamini-Hochberg correction. Asterisks indicate significant differences *p<0.05, **p<0.01, ***p<0.001.

*Supplement Figure 6. Disruption of microbial rhythms in Bmal1^IEC-/-^xIL-10^-/-^ mice*

(A) Beta-diversity illustrated by MDS plots of fecal microbiota based on generalized UniFrac distances (GUniFrac) in control, *IL-10*^-/-Sv129^ and *Bmal1*^IEC-/-^x*IL-10*^-/-^ mice under AD condition. (B) Normalized richness and (C) abundance of major phyla (*Bacteroidota* and *Firmicutes*) in control, *IL-10*^-/-BL6^ and *Bmal1*^IEC-/-^x*IL-10*^-/-^ mice under AD condition. (D) Heatmap depicting the relative abundance of identified zOTUs (mean relative abundance>0.1%; prevalence > 10%). Data from control, *IL-10*^-/-BL6^ and *Bmal1*^IEC-/-^x*IL-10*^-/-^ mice are normalized to the peak of each zOTU and ordered by the peak phase in control group. Yellow means high abundance and blue low abundance. (E) Manhattan plot of the amplitude and adj.p value of zOTUs identified in control, *IL-10*^-/-BL6^ and *Bmal1*^IEC-/-^x*IL-10*^-/-^ mice. (F) Clock genes (*Reverba*, *Cry1)* expression measured at CT13 in colon of control, *IL-10*^-/-BL6^ and *Bmal1*^IEC-/-^x *IL-10*^-/-^ mice under AD and RF conditions. (G) Beta-diversity illustrated by MDS plots of fecal microbiota based on generalized UniFrac distances (GUniFrac) in control, *IL-10*^-/-Sv129^ and *Bmal1*^IEC-/-^x*IL-10*^-/-^ mice (top) and normalized richness (bottom) under RF condition. (H) Taxonomic tree of fecal microbiota which have restored rhythms in *IL-10*^-/-^ mice after RF but not in *Bmal1*^IEC-/-^x*IL-10*^-/-^ mice after RF. Taxonomic ranks are from phylum (outer dashed ring), family (inner ring highlighted) to genera (middle, color coded according to family) which are indicated by the individual branches. Data points represent individual mice (n>6/time points for each group). Significant rhythms are illustrated with fitted cosine-regression; data points connected by straight lines indicate no significant cosine fit curves (p > 0.05) and thus no rhythmicity. Data are represented as mean ± SEM. Statistics were performed by three-way ANOVA following Benjamini, Krieger and Yekutieli correction correction. Asterisks indicate significant differences *p<0.05, **p<0.01, ***p<0.001, ****p<0.0001
