# Supplementary material for: Targeting the intestinal circadian clock by meal timing ameliorates gastrointestinal inflammation
Source: Cell Mol Immunol. 2024 Jun 25;21(8):842–55. doi: 10.1038/s41423-024-01189-z (PMC11291886; doi:10.1038/s41423-024-01189-z)
Supplement: Supplementary file 10 — Supplemental Table 3 [file 41423_2024_1189_MOESM10_ESM.pdf]

SupplementalTable 3 Statistical comparison between IL-10-/- AD and IL-10-/- RF groups

**2 way ANOVA comparison for group *IL-10* $^{-/-}$ AD vs *IL-10* $^{-/-}$ RF**								
Fig. 2B								
Time points	1	4	7	10	13	16	19	22
Significance	Group differences p=0.0004							
Fig. 2C								
Bacteroidota	Group differences p<0.0001							
Firmicutes	Group differences p<0.0001							
Fig. 2E								
Lachnospiraceae	Group differences p<0.0001							
Oscillospiraceae	Group differences p=0.0005							
zOTU128	Group differences p=0.0112							
zOTU130	Group differences p=0.0411							
