# Supplementary material for: Targeting the intestinal circadian clock by meal timing ameliorates gastrointestinal inflammation
Source: Cell Mol Immunol. 2024 Jun 25;21(8):842–55. doi: 10.1038/s41423-024-01189-z (PMC11291886; doi:10.1038/s41423-024-01189-z)
Supplement: Supplementary file 13 — Supplemental Table 6 [file 41423_2024_1189_MOESM13_ESM.pdf]

Supplemental Table 6: Summary of sample size of all experiments for all Figures

WTAD: control group under ad libitum **WTRF**: control group under restricted feeding **KOAD**: IL-10-/- group under ad libitum **KORF**: IL-10-/- under restricted feeding

Fig.1B-E	WTAD	WTRF	KOAD	KORF
n (6 time points)	4,5,4,4,4,4	4,4,4,4,4,4	4,4,4,4,4,4	3,2,4,2,4,4
Fig.2B-E	WTAD	WTRF	KOAD	KORF
n (8 time points)	6,5,5,4,5,6,5,5	7,9,9,7,8,8,9,8	3,4,5,5,5,4,5,4	6,7,9,9,7,9,8,9
Fig.2G	WTAD	KOAD		
n (8 time points)	6,5,5,4,5,6,5,5	3,4,5,5,5,4,5,4		
Fig.2G-H	WTAD	KOAD		
n (8 time points)	3,3,3,3,3,3,3,3	6,6,6,6,6,6,6,6		
Fig.4	Control	Bmal1IEC-/-		
n (6 time points)	4,4,4,4,4,4	4,4,4,4,4,4		

Fig.3, Fig.5-6 Data are illustrated as single dot and each dot represents one mouse
