# Supplementary material for: Targeting the intestinal circadian clock by meal timing ameliorates gastrointestinal inflammation
Source: Cell Mol Immunol. 2024 Jun 25;21(8):842–55. doi: 10.1038/s41423-024-01189-z (PMC11291886; doi:10.1038/s41423-024-01189-z)
Supplement: Supplementary file 14 — Supplemental Material [file 41423_2024_1189_MOESM14_ESM.docx]

METHODS

# **Ethics Statement**

Experiments were conducted at Technical University of Munich in accordance with Bavarian Animal Care and Use Committee (TVA ROB-55.2Vet-2532.Vet_02-18-14).

# **Mouse models**

## *Bmal1*^IEC -/-^ and *Bmal1*^flox/flox^ mouse generation

Male intestinal epithelial cell-specific *Bmal1* knock-out *(Bmal1fl/fl* x Villin CRE/wt; referred to as *Bmal1*^IEC-/^*^-^*) mice and their control littermates (*Bmal1fl/fl* x Villin wt/wt ; referred to as *Bmal1*^flox/flox^) on a genetic C57BL/6J background were generated as previously described^1^. Breeding was performed by crossing *Bmal1fl/fl* x Villin CRE/wt with *Bmal1fl/fl* x Villin wt/wt. Mice were kept in LD 12:12 cycles (300 lux), with lights turned on at 5am (*Zeitgeber* time (ZT0)) to 5pm (ZT12)) unless stated otherwise. At the age of 8 weeks, mice were single housed with running wheel at 22  ±  1 °C. Mice have ad libitum access to chow diet (V1124-300, Ssniff Diets, Soest, Germany) and water under specific-pathogen free (SPF) conditions according the FELASA recommendation unless stated otherwise.

*Bmal1*^IEC -/-^x*IL-10*^-/-BL6^ mouse generation and *IL-10*^-/-Sv129^ mouse

Male intestinal epithelial cell-specific *Bmal1* and interleukin-10 double knock-out mice (*Bmal1*^IEC -/-^x*IL-10*^-/-BL6^) were generated by crossing *Bmal1*^flox/flox^x*IL-10*^+/-BL6^ with *Bmal1*^IEC-/-^x*IL-10*^+/-BL6^ under SPF conditions and bred for several generations internally to harmonize the intestinal microbiota. Interleukin-10 knock-out mice under BL6 and Sv129 background were initially provided by The Jackson Laboratory and bred internally.

# Tissue collection

# All male mice, unless stated otherwise, were sacrificed by cervical dislocation at the age of 18-20 weeks in the second day of darkness at the indicated circadian times (CT), which is considered as the same indicated time points used for normal LD. This was performed independent of external timing cues (Zeitgeber), such as the light-dark cycle to demonstrate rhythms generated by endogenous intestinal clocks ^3^. Eyes were removed prior to tissue dissection in dim red light. Tissues were harvested and directly transferred into RNA stabilization solution (NucleoProtect^®^ RNA, MACHEREY-NAGEL) overnight at 4 degrees and then stored in -80 degrees. For fixation, tissues were freshly harvested and then transferred into 4% Formaldehyde.

**Organoid**

Freshly isolated tissue pieces from small intestine and colon were placed in phosphate-buffered saline (PBS) and 120µl 0.5M EDTA to detach the villi from the tissue. Tissues were changed to new tubes after 30 min incubation at 4 °C with gently shaking. Intestinal crypts were resuspended in Matrigel™ (BD Biosciences) and then seeded in 25 μl drops in 64-well plates after filtering and centrifuging. Plates were incubated at 37ºC for 15min to allow suspension to polymerize before fresh medium was supplied. Organoid medium (IntesticultTM, Stemcell) was replaced every 3-4 days. Organoids for each time point were plated into a separate plate to limit manipulation or exposure to possible resetting cues, such as temperature. After synchronization with serum shock (50% Fetal Bovine Serum and 50% Intesticult) for 2 hours, medium was replaced with basic organoid medium (IntesticultTM, Stemcell). Every 6 hours over a 24-hour period (starting at 12h after serum shock) medium was removed and organoids were transferred to -80°C until further processing.

Gene expression analysis

RNA was extracted according to the manufacturer’s instructions (NucleoSpin^®^ RNA, MACHEREY-NAGEL) and measured by NanoPhotometer^®^N60 (IMPLEN). cDNA was synthesized from 1000ng RNA using cDNA synthesis kit Multiscribe RT (Thermofischer Scientific). qPCR was performed in a Light Cylcer 480 system (Roche Diagnostiscs, Mannheim, Germany) using Universal Probe Library system according to manufacturer’s instructions. Calculations (2^–ΔΔCt^ method) were normalized to elongation factor 1 alpha as housekeeper. For genes expression the following primers and probes were used: Brain and Muscle ARNT-Like 1 (*Bmal1*) F 5’-ATTCCAGGGGGAACCAGA-3’ R 5’-GGCGATGACCCTCTTATCC-3’ Probe 15, Period 2 (*Per2*) F 5’-TCCGAGTATATCGTGAAGAACG-3’ R 5’- CAGGATCTTCCCAGAAACCA-3’ probe 5, Nuclear receptor subfamily 1 group D member 1 (*Reverbα*) F 5’-AGGAGCTGGGCCTATTCAC-3’ R 5’-CGGTTCTTCAGCACCAGAG-3’ probe 1, D site-binding protein (*Dbp*) F 5’-ACAGCAAGCCCAAAGAACC-3’ R 5’- GAGGGCAGAGTTGCCTTG-3’ probe 94, Cryptochrome 1 (*Cry1*) F 5’- ATCGTGCGCATTTCACATAC-3’ R 5’-TCCGCCATTGAGTTCTATGAT-3’ probe 85, Tumor necrosis factor alpha (*Tnf*) F 5’-TGCCTATGTCTCAGCCTCTTC-3’ R 5’-GAGGCCATTTGGGAACTTCT-3’ probe 49, Interferon gamma (IFN-γ) F 5’-GGAGGAACTGGCAAAAGGAT-3’ R 5’-TTCAAGACTTCAAAGAGTCTGAGG-3’ probe 21, RNA abundance was normalized to the housekeeping gene Elongation factor 1-alpha (*Ef1a*) F 5’-GCCAAT TTCTGGTTGGAATG-3’ R 5’-GGTGACTTTCCATCCCTTGA-3’ probe 67. SYBR (SensiFAST SYBR No-ROX kit, Meridian Bioscience) was used to measure genes Chemokine (C-X-C motif) receptor 4 (*Cxcr4*) F 5’-GACTGGCATAGTCGGCAATGGA-3’ R 5’-CAAAGAGGAGGTCAGCCACTGA-3’ and Vascular cell adhesion molecule 1 (*Vcam-1*) F 5’- GCTATGAGGATGGAAGACTCTGG-3’ R 5’- ACTTGTGCAGCCACCTGAGATC-3’.

# **High-Throughput 16S Ribosomal RNA (rRNA) Gene Amplicon Sequencing Analysis**

Genomic DNA was isolated from snap-frozen fecal pellets and sequenced as previously described^7, 8^. Briefly, DNA NucleoSpin gDNA columns (Machery-Nagel, No. 740230.250) were used for DNA purification. After amplification of the V3-V4 region of the 16S rRNA gene, the multiplexed samples were sequenced on an Illumina HiSeq in paired-end mode (2x250 bp) using the Rapid v2 chemistry. Two negative controls, consisting of DNA stabilizer without stool, were used for every 45 samples to control for artifacts and insure reproducibility. High-Quality sequences of read counts > 5000 were used for 16s rRNA data analysis. Reads FASTQ files were consequently processed using an in-house developed NGSToolkit (Version Toolkit 3.5.2_64 ) based on USEARCH 11^9^. After trimming and FASTQ quality check, quality filtered reads were merged, deduplicated, clustered and a denoised clustering approach was applied to generate zOTUs^10^. Taxonomic assignment was performed with the EZBiocloud database^11^. Data was further analyzed with the R-based pipeline RHEA^12^. Phylogenetic trees are generated through the software MegaX^13^. Trees were visualized and annotated with the use of the online tool EvolView (<http://www.evolgenius.info/evolview/>)^14^. Comparison of genotype and treatment differences were performed by SIAMCAT^15^.

# **Targeted metabolite analyses**

## *Sample preparation for targeted metabolite analyses*

Approximately 20 mg of mouse cecal content was weighed extracted by bead beating (3 times of 20s 6 m/s with 30s breaks) with FastPrep-24 5G bead beating grinder (MP Biomedicals) supplied with a CoolPrep adapter. To measure BA and SCFAs, we used multiple reaction monitoring method and 3-NPH method respectively, as described previously^16^. Analyst 1.7 software (Sciex, Darmstadt, Germany) were used for data acquisition.

## *Targeted bile acid measurement*

Targeted bile acid measurement was performed as previously descrived^7^. Briefly, measurement was performed using a QTRAP 5500 triple quadrupole mass spectrometer (Sciex, Darmstadt, Germany) coupled to an ExionLC AD (Sciex, Darmstadt, Germany) ultrahigh performance liquid chromatography system. The MS parameters and LC conditions were optimized using commercially available standards of endogenous bile acids and deuterated bile acids, for the simultaneous quantification of selected 44 analytes. Data acquisition and instrumental control were performed with Analyst 1.7 software (Sciex, Darmstadt, Germany) as previously described^16^.

## *Targeted short-chain fatty acid measurement*

As previously described^7^, in brief, 40 µL of the cecal extract and 15 µL of isotopically labeled standards (ca 50 µM) were mixed with 20 µL 120 mM EDC HCl-6% pyridine-solution and 20 µL of 200 mM 3-NPH HCL solution. The measurement system was the same as described above. Data acquisition and instrumental control were performed with Analyst 1.7 software (Sciex, Darmstadt, Germany).

**Untargeted metabolite analyses**

Fecal samples were collected from *IL-10*^-/-Sv129^ mice and their littermates wild types every 3 hours over the course of a 24h day. Samples were directly snap frozen and stored at -80 ◦C upon metabolite extraction. The untargeted analysis was performed using a Nexera UHPLC system (Shimadzu, Duisburg, Germany) coupled to a Q-TOF mass spectrometer (TripleTOF 6600, AB Sciex, Darmstadt, Germany). Separation of the fecal samples was performed either using a UPLC BEH Amide 2.1 × 100 mm, 1.7 µm analytic column (Waters, Eschborn, Germany) with a 400 µL/min flow rate or with a Kinetex XB18 2.1 x 100 mm, 1.7 µm (Phenomenex, Aschaffenburg, Germany) with a 300 µL/min flow rate. For the HILIC-separation the settings were as follows: The mobile phase was 5 mM ammonium acetate in water (eluent A) and 5 mM ammonium acetate in acetonitrile/water (95/5, v/v) (eluent B). The gradient profile was 100% B from 0 to 1.5 min, 60% B at 8 min and 20% B at 10 min to 11.5 min and 100% B at 12 to 15 min. For the reversed-phase separation eluent A was 0.1% formic acid and eluent B was 0.1% formic acid in acetonitrile. The gradient profile started with 0.2% B which was held for 0.5 min. Afterwards the concentration of eluent B was increased to 100% until 10 min which was held for 3.25 min. Afterward the column was equilibrated at starting conditions. A volume of 5 µL per sample was injected. The autosampler was cooled to 10 °C and the column oven heated to 40 °C. Every tenth run a quality control (QC) sample which was pooled from all samples was injected. The samples were measured in a randomized order and in the Information Dependent Acquisition (IDA) mode. MS settings in the positive mode were as follows: Gas 1 55, Gas 2 65, Curtain gas 35, Temperature 500 °C, Ion Spray Voltage 5500, declustering potential 80. The mass range of the TOF MS and MS/MS scans were 50–2000 m/z and the collision energy was ramped from 15–55 V. MS settings in the negative mode were as follows: Gas 1 55, Gas 2 65, Cur 35, Temperature 500 °C, Ion Spray Voltage –4500, declustering potential –80. The mass range of the TOF MS and MS/MS scans were 50–2000 m/z and the collision energy was ramped from –15–55 V.

The “msconvert” from ProteoWizard^17^ were used to convert raw files to mzXML (de-noised by centroid peaks). The bioconductor/R package xcms^18^ was used for data processing and feature identification. More specifically, the matched filter algorithm was used to identify peaks (full width at half maximum set to 7.5 s). Then the peaks were grouped into features using the “peak density” method^18^. The area under the peaks was integrated to represent the abundance of features. The retention time was adjusted based on the peak groups presented in most of the samples. To annotate possible metabolites to identified features, the exact mass and MS2 fragmentation pattern of the measured features were compared to the records in HMDB^19^ and the public MS/MS database in MSDIAL^20^, referred to as MS1 and MS2 annotation, respectively. The QC samples were used to control and remove the potential batch effect, t-test was used to compare the features’ intensity between the groups.

The associated untargeted metabolomics data are available at <https://massive.ucsd.edu/ProteoSAFe/dataset.jsp?task=ea2d927ea408493ea65d4fae9354ce20>

**Transfer experiment**

Cecal microbiota (collected at CT13) from either *Bmal1^IEC-/-^* and their controls, or severely inflamed *IL-10*^-/-BL6^ mice (n=4, mixture) were gavaged into germ-free *IL-10*^-/-BL6^ recipient mice, or germ-free *Bmal1^IEC-/-^* and their control recipient mice, respectively. 100µl of 7x10^6^ bacteria/µl were used for gavaging each mouse. Mice were weekly monitored for bodyweight changes and feces was sampled and stored in DNA stabilizer at week 5 after gavage. Mice were sacrificed on the second day of constant darkness at CT1 and CT13. All mice were kept in the gnotobiology facility in isolators equipped with HEPA-filters at 22 ± 1 °C with a 12-h light/dark cycle (lights on 5 am till 5 pm). Mice were single housed and had ad libitum access to autoclaved chow (V1124-300, Sniff Diets, Soest, Germany) and autoclaved water.

## Immune cell isolation from the lamina propria

Freshly isolated intestinal tissues were flipped, rinsed and cut into 1 cm pieces. After 15 min incubation and shaking in DMEM with 20 µL of 1M DTT, tissue pieces were transferred into 37°C PBS containing 200 µL of 150mM EDTA for 10 min shaking. Jejunum tissue was then digested at 37°C for approximately 15 min in Thermoshake (Gerhardt) with 0.6 mg/ml type VIII collagenase (Sigma-Aldrich). Colonic tissue was digested at 37°C for approximately 25 min with 0.85 mg/ml type V collagenase (Sigma-Aldrich), 1.25 mg/ml collagenase D (Sigma-Aldrich), 10µl/ml Amphotericin (100x), 1 mg/mL Dispase II, and 10 U/µL DNase-V (Sigma). Following digestion, cells were passed through a 40 µm strainer and washed with PBS. Consequently, cells were fixed with 2% PFA for 20 min and washed, and stored in RPMI at 4 °C until further processing.

## Flow cytometry measurement

For intracellular stainings, cells were permeabilized with 0.5% saponin and stained with conjugated antibodies at dilution 1/100-1/50 for 30 min. Namely, anti CD8-PE, anti CD3 -PerCP/Cy5.5, anti CD4-FITC, anti IL-17a -PE/cy7, anti INFy- APC.Surface stainings were performed using anti CD11c-PE, anti CD11b-APC/Cy7, anti F4/80-PE/cy7, anti Ly6G-APC conjugated antibodies at dilution 1/50 for 30 min. Cells were washed and resuspended and passed through Invitrogen™ Attune™ NxT Flow Cytometer. Analysis was performed using FlowJo v10.7.2.

## Histology

Fixated and dehydrated tissue sections were cut into 5 μm thick slices and consequently stained according to the following steps: xylene/ 5 min, xylene/ 5 min, Ethanol 100%/ 5 min, Ethanol 100%/ 5 min, Ethanol 96%/ 2 min, Ethanol 96%/ 2 min, Ethanol 70%/ 2 min, Ethanol 70%/ 2 min, Water/ 30 s, hematoxylin/ 2 min, tap water/ 15 s, Scotts Tap Water/ 30 s, Water/ 30 s, Ethanol 96%/ 30 s, Eosin/ 30 s, Ethanol 96%/ 30s, Ethanol 96%/ 30 s, Ethanol 100%/ 30 s, Ethanol 100%/ 30 s, Xylene/ 90 s, Xylene/ 90s (Leica ST5020 multistainer). DPX new mounting media (Merck) was added to preserve the tissues. Histological scores were assessed blindly based on the degree of immune cell infiltration of all colonic wall layers (mucosa, submucosa and muscularis), crypt hyperplasia, goblet cell depletion and mucosal damage, resulting in a score from 0 (not inflamed) to 12 (severely inflamed) according to Katakura method (Katakura, Lee et al. 2005).

For AB/PAS staining, tissue slices were deparaffinized and rehydrated before being stained with Alcian blue solution for acidic mucins (1% volume/volume in 3% acetic acid, pH 2.5, 15 minutes), treated with periodic acid solution (0.5% volume/volume, 5 minutes) and co-stained with Schiff’s reagent for neutral mucins (Sigma-Aldrich, 10 minutes). Nuclei were then counterstained with hematoxylin. Consequently, tissue sections were differentiated (0.2% ammonia water), dehydrated, and mounted. The number of goblet cells was calculated as a total number per 100 μm2.

### References

1. Kawai M, Kinoshita S, Yamazaki M, et al. Intestinal clock system regulates skeletal homeostasis. JCI Insight 2019;4.

2. Jud C, Schmutz I, Hampp G, et al. A guideline for analyzing circadian wheel-running behavior in rodents under different lighting conditions. Biol Proced Online 2005;7:101-16.

3. Aschoff J. Exogenous and endogenous components in circadian rhythms. Cold Spring Harb Symp Quant Biol 1960;25:11-28.

4. Wu T, Hu E, Xu S, et al. clusterProfiler 4.0: A universal enrichment tool for interpreting omics data. Innovation (Camb) 2021;2:100141.

5. Wu G, Anafi RC, Hughes ME, et al. MetaCycle: an integrated R package to evaluate periodicity in large scale data. Bioinformatics 2016;32:3351-3353.

6. Pelikan A, Herzel H, Kramer A, et al. Venn diagram analysis overestimates the extent of circadian rhythm reprogramming. FEBS J 2022;289:6605-6621.

7. Heddes M, Altaha B, Niu Y, et al. The intestinal clock drives the microbiome to maintain gastrointestinal homeostasis. Nat Commun 2022;13:6068.

8. Reitmeier S, Kiessling S, Clavel T, et al. Arrhythmic Gut Microbiome Signatures Predict Risk of Type 2 Diabetes. Cell Host Microbe 2020;28:258-272 e6.

9. Edgar RC. Search and clustering orders of magnitude faster than BLAST. Bioinformatics 2010;26:2460-1.

10. Edgar RC. UNOISE2: improved error-correction for Illumina 16S and ITS amplicon sequencing. bioRxiv 2016:081257.

11. Yoon SH, Ha SM, Kwon S, et al. Introducing EzBioCloud: a taxonomically united database of 16S rRNA gene sequences and whole-genome assemblies. Int J Syst Evol Microbiol 2017;67:1613-1617.

12. Lagkouvardos I, Fischer S, Kumar N, et al. Rhea: a transparent and modular R pipeline for microbial profiling based on 16S rRNA gene amplicons. PeerJ 2017;5:e2836.

13. Kumar S, Stecher G, Li M, et al. MEGA X: Molecular Evolutionary Genetics Analysis across Computing Platforms. Mol Biol Evol 2018;35:1547-1549.

14. Subramanian B, Gao S, Lercher MJ, et al. Evolview v3: a webserver for visualization, annotation, and management of phylogenetic trees. Nucleic Acids Res 2019;47:W270-W275.

15. Wirbel J, Zych K, Essex M, et al. Microbiome meta-analysis and cross-disease comparison enabled by the SIAMCAT machine learning toolbox. Genome Biol 2021;22:93.

16. Reiter S, Dunkel A, Metwaly A, et al. Development of a Highly Sensitive Ultra-High-Performance Liquid Chromatography Coupled to Electrospray Ionization Tandem Mass Spectrometry Quantitation Method for Fecal Bile Acids and Application on Crohn's Disease Studies. J Agric Food Chem 2021;69:5238-5251.

17. Kessner D, Chambers M, Burke R, et al. ProteoWizard: open source software for rapid proteomics tools development. Bioinformatics 2008;24:2534-6.

18. Smith CA, Want EJ, O'Maille G, et al. XCMS: processing mass spectrometry data for metabolite profiling using nonlinear peak alignment, matching, and identification. Anal Chem 2006;78:779-87.

19. Wishart DS, Feunang YD, Marcu A, et al. HMDB 4.0: the human metabolome database for 2018. Nucleic Acids Res 2018;46:D608-D617.

20. Tsugawa H, Cajka T, Kind T, et al. MS-DIAL: data-independent MS/MS deconvolution for comprehensive metabolome analysis. Nat Methods 2015;12:523-6.

21. Douglas GM, Maffei VJ, Zaneveld JR, et al. PICRUSt2 for prediction of metagenome functions. Nat Biotechnol 2020;38:685-688.

22. Hughes ME, Hogenesch JB, Kornacker K. JTK_CYCLE: an efficient nonparametric algorithm for detecting rhythmic components in genome-scale data sets. J Biol Rhythms 2010;25:372-80.
